# Effects of tetrahydroindenoindole supplementation on metabolism: A systematic review with meta-analysis of rodent-based studies

**DOI:** 10.1007/s11357-025-01680-z

**Published:** 2025-05-05

**Authors:** Miguel Pérez-Rodríguez, Rafael A. Casuso, Sandra Rodríguez-López, José A. González-Reyes, José M. Villalba

**Affiliations:** 1 Department of Cell Biology, Physiology and Immunology, University of Córdoba, and ceiA3 Campus of International Excellence in Agrifood, Córdoba, Spain; 2 Department of Health Sciences, Universidad Loyola Andalucía, Córdoba, Spain; 3 Laboratory of Integrative Systems Physiology, Institute of Bioengineering, École Polytechnique Fédérale de Lausanne, Lausanne, Switzerland

**Keywords:** Meta-analysis, Metabolism, Mice, Systematic review, Rodents, Tetrahydroindenoindole

## Abstract

Identifying novel compounds with therapeutic potential is a main area of interest in biomedical research. Tetrahydroindenoindole (THII) has emerged as a compound of interest due to both its antioxidant properties and its action as a pharmacological activator of the enzyme cytochrome *b*_5_ reductase 3. However, there is a lack of comprehensive synthesis of findings, particularly concerning the effects of THII on metabolism in mice under non-disease conditions. This systematic review with meta-analysis aims to bridge this gap by analyzing existing studies. Our findings demonstrated that THII supplementation reduced body weight gain, while fat mass remained unchanged. Fasting blood glucose and plasma insulin levels, as well as insulin levels during the glucose tolerance test, showed no changes. However, glucose tolerance improved with THII supplementation during the glucose tolerance test, particularly in animals under a high-fat diet. THII supplementation also increased O_2_ consumption and CO_2_ production, with a tendency to lower respiratory quotient. In mitochondria, THII supplementation did not affect state 3 respiration, while increased state 4, and decreased the respiratory exchange ratio. Notably, mitochondrial H_2_O_2_ production during state 4 respiration and ATP levels also remained unchanged. Furthermore, THII supplementation reduced NADPH-dependent O_2_ uptake, NADPH-dependent H_2_O_2_ production, and lipid peroxidation. Despite the limitations and potential sources of bias identified, we observed valuable outcomes linked to THII supplementation. The significant impact on energy metabolism, mitochondrial function, and oxidative stress underscores THII as a promising intervention with translational relevance for aging-related alterations, enhancing healthspan, and targeting metabolic-associated diseases such as obesity or diabetes.

**Supplementary Information:**

The online version contains supplementary material available at 10.1007/s11357-025-01680-z.

## Introduction

The discovery of novel compounds with therapeutic potential is a cornerstone of biomedical research. Among these, tetrahydroindenoindole (THII) has gained particular attention due to its antioxidant properties and its significant association with the enzyme cytochrome *b*_*5*_ reductase 3 (CYB5R3). THII is a potent antioxidant that acts through electron-mediated reductive scavenging of free radicals, suggesting its potential to mitigate oxidative stress-related metabolic disorders [1]. CYB5R3 plays a critical role in processes such as fatty acid elongation and desaturation, cholesterol biosynthesis, and drug metabolism, and its overexpression has been linked to enhanced healthspan and extended lifespan [2]. Recent studies have identified THII as a pharmacological activator of CYB5R3, with evidence showing that the effects of THII are mediated through the activation of this enzyme [2–5].

Oxidative stress, which occurs when there is an imbalance between reactive oxygen species (ROS) production and the antioxidant defenses, is a key factor in the development of aging and aging-related diseases, such as cancer, diabetes, neurodegenerative diseases or cardiovascular disorders [6]. This imbalance can result in cellular damage, inflammation, and disrupted metabolic functions [7]. Antioxidant compounds such as THII, which neutralize ROS and modulate metabolic enzymes like CYB5R3, offer promising therapeutic approaches for managing these conditions.

The metabolic effects of THII have primarily been studied in animal models, particularly mice due to their genetic and physiological similarities to humans and well-characterized metabolic pathways. Thus, THII has been evaluated in various rodent models of disease, including hepatotoxicity [8], epidermal tumor formation [9], type I and type II diabetes [10], 2,3,7,8-tetrachlorodibenzo-p-dioxin toxicity [11], olanzapine-induced metabolic dysfunction [12], and sulfonylureas treatment secondary failure [3, 4]. Preliminary findings suggest that THII supplementation may influence key metabolic processes, including glucose homeostasis, energy metabolism, and ROS management [10–12]. However, a comprehensive synthesis of these findings on the overall effects of THII supplementation on mouse metabolism remains lacking, particularly under non-pathological conditions.

This systematic review with meta-analysis aims to address this gap by evaluating the existing scientific literature in the field. By synthesizing data from the available studies, we seek to clarify the consistency of findings across different experimental settings and uncover potential mechanisms driving the effects of THII. This analysis will not only enhance our understanding of the role of THII in metabolism, but also may provide insights into its therapeutic potential and guide future research.

Besides exploring the therapeutic potential of THII, this systematic review will critically evaluate the methodologies and quality of evidence available. By identifying areas of consensus, pinpointing knowledge gaps, and highlighting methodological challenges, we aim to establish a robust basis for further investigations. Ultimately, this work aspires to advance the understanding of THII as a metabolic modulator and lead to the development of more effective interventions targeting metabolic health.

## Methods

### Data search and sources

The Medline (via PubMed), Web of Science, and Scopus databases were systematically reviewed to identify relevant studies up to May 29, 2024, with an updated search conducted on March 31, 2025. Studies analyzing the effects of THII on mice metabolism were included. The search strategy used the terms: ("indenoindole" OR "tetrahydroindenoindole" OR "THII"). This systematic review was prospectively registered in the International Prospective Register of Systematic Reviews (PROSPERO) under registration number CRD42024475369.

### Study selection

The initial search yielded a total of 490 studies from the Medline (via PubMed), Web of Science, and Scopus databases. After removing duplicates, 277 unique studies remained. Titles and abstracts of these studies were independently screened by two reviewers (R.A.C. and M.P.) to assess their relevance based on predefined inclusion and exclusion criteria. From this screening, 11 potentially relevant studies were identified, and their full-text articles were retrieved for further evaluation. Additionally, related articles and studies citing these potentially relevant works were examined, leading to the identification of 2 additional articles of interest. Ultimately, 7 studies met the inclusion criteria and were included in the qualitative analysis, with 5 of these studies also being included in the quantitative analysis (Suppl. Figure 1).

Studies including an experimental group targeting the effects of THII administration on rodent metabolism were selected. Specific inclusion criteria required in vivo studies using standardized normal diet (NDI) and/or high-fat diet (HFD). Exclusion criteria included review articles, in vitro cellular models, or studies using non-rodent animal models. In cases where study abstracts mentioned THII supplementation alongside other interventions (e.g., drugs or transgenesis), full-text evaluations were conducted to ensure that the control group met the inclusion criteria, as the effects of THII in combination with other compounds or in the context of genetically modified models were not considered. Any discrepancies during the selection process were resolved through discussion and consensus between the reviewers.

### Data extraction, synthesis, analysis, and visualization

The following data were extracted by two researchers (R.A.C. and M.P.) independently: number, sex, age, and strain of animals; length of the intervention and dosage; experimental condition (control/standard diet-fed animals or HFD-fed animals); and any reported marker related to metabolism. The extracted metabolic data included body weight, body weight gain, body fat percentage, glucose levels, insulin levels, oxygen consumption, CO_2_ production, mitochondrial respiration, ATP production, ROS production, and lipid peroxidation, along with other specific outcomes of interest for particular studies, such as liver detoxification enzyme levels, lifespan, or *Cyb5r3* transcripts levels. The results for all these parameters from the selected studies were included in the qualitative analysis. However, only those parameters with consistently reported results across the different studies were considered for the quantitative analysis. In the latter case, values for each parameter were recorded as means for both the control and experimental conditions under THII supplementation at the final time point of the intervention, along with their corresponding standard deviations (SD). In cases where values were reported as the standard error of the mean (SEM), they were converted to SD. When not provided in the text or tables, data were extracted using WebPlotDigitalizer [13, 14]. Any discrepancies between reviewers were resolved by consensus.

All analyses were performed using the metafor package (v4.4–0) [15] for R software (v4.2.3) [16]. The meta-analyses were performed using random-effects models with DerSimonian–Laird methods to assess the effect of THII supplementation on mice metabolism. Heterogeneity between studies was assessed using Cochran’s Q and I^2^ statistics. Effect sizes are presented as standardized mean differences (SMDs) and 95% CIs, as the outcomes have noncomparable scales. We performed a subgroup analysis exploring the difference between experimental conditions (NDI *vs*. HFD), tissues, or protocol conditions (substrates). Subgroup analyses were performed only when a minimum of two studies were available within that subgroup. A *p*-value threshold of < 0.05 was considered statistically significant, while p-values between 0.05 and 0.1 were interpreted as indicative of tendency. The results were represented using forest plots.

### Risk of bias

The risk of bias and study quality were assessed using two complementary approaches: the risk of bias tool developed by the Systematic Review Centre for Laboratory Animal Experimentation (SYRCLE) [17] and the checklist designed by the Collaborative Approach to Meta-Analysis and Review of Animal Data from Experimental Studies (CAMARADES) [18]. R.A.C. and M.P. independently conducted the quality assessment of the included animal studies. SYRCLE's risk of bias tool, which consists of 10 items, was used to evaluate internal validity by assessing selection bias, performance bias, detection bias, attrition bias, reporting bias, and other sources of bias. Each item was scored as low, high, or unclear risk of bias. A maximum score of 1 was assigned to each item: 1 for low risk of bias, 0 for high risk of bias, and 0.5 for unclear risk of bias. If an evaluated item included subitems, each subitem was evaluated independently, and a summarized score for the item was calculated as a weighted average based on the scores of its subitems. Additionally, an adaptation of CAMARADES checklist was used to assess both internal and external validity, as well as overall study quality. This 10-item checklist included criteria such as publication in a peer-reviewed journal, statement of temperature control, random allocation to treatment or control, blinded assessment of outcome, sample size calculation, compliance with animal welfare regulations, statement of potential conflict of interest, and others. Each item was scored as either present (1) or absent (0). The overall risk of bias evaluated by the two approaches described above was calculated as a total score, with a maximum possible score of 10. Studies were categorized as having a low risk of bias if they scored > 7.5, moderate risk if they scored between 5–7.5, and high risk if they scored < 5. Any discrepancies in scoring between the two reviewers were resolved through discussion and consensus. When consensus could not be reached, disagreements were resolved by consulting S.R., a third reviewer.

Publication bias was assessed using Egger's test through the metafor package (v4.4–0) in R (v4.2.3) [15], and the results were visualized using funnel plots. Additionally, multiple sensitivity analyses were conducted using a leave-one-out approach implemented through the metafor package (v4.4–0) in R (v4.2.3), to determine whether the results were influenced by the exclusion of individual studies. In the sensitivity analyses, studies were excluded one at a time, and the results were reanalyzed for each exclusion, enabling the evaluation of the impact of each individual study on the final outcome. These analyses were planned, performed systematically, and were not influenced by the results obtained during the analysis.

## Results

### Study characteristics

Table 1 shows the results from the qualitative analysis, summarizing key details from the seven studies included, such as the THII dose, intervention duration, animal model, and the sex and age of the animals. It also includes the type of diet followed by the animals (NDI or HFD), the number of animals included in each study, and the main reported outcomes.

**Table 1:** Qualitative analysis of the selected studies summarizing key details. Reference (Ref.), “Study” and “Journal” columns identify the articles included in the meta-analysis

Ref	Study	Journal	Given dose	Actual intake	Time	Animal model	Sex	Age	Diet	N. of animals	Results (increased markers)	Results (reduced markers)	Results (unchanged markers)
[23]	Shertzer and Sainsbury, 1991	Fd Chem Toxic	50 mg/kg of weight/10 days (gavage)	5 mg/kg/day	10 days	Wistar rat	Male	NA	NDI	6	EROD levels	No parameter showed an increase	Liver weight; amount of liver DNA and microsomal proteins; levels of cytochrome P-450, aminopyrine N-demethylase, NDMA N-demethylase, ascorbate synthase, NADPH cytochrome c reductase, UDPGT, GST, GSSG-Red, GSH-Px, quinone reductase, and SOD
[10]	Shertzer et al., 2009	Chem Biol Interact	100 µM (water)	4.5 mg/kg/day	10 weeks	C57BL/6 mice	Female	8–10 weeks	NDI	8	O_2_ consumption; CO_2_ production	RQ; H_2_O_2_ production and RCR in liver; O_2_ uptake and H_2_O_2_ production using NADPH in WAT	Body weight; weight gain; body fat; fasting blood glucose; glucose tolerance; ATP production in liver; O_2_ uptake and H_2_O_2_ production under mitochondrial state 4 in WAT; H_2_O_2_ production using NADPH; 4-HAE and MDA levels
									HFD	4	No parameter showed an increase	H_2_O_2_ production in liver, and O_2_ uptake and H_2_O_2_ production using NADPH or NADPH + DPI as substrate in WAT, also 4-HAE levels	Weight gain; body fat; fasting blood glucose; mitochondrial state 3 and 4 respiration, RCR, and ATP levels in liver; O_2_ uptake and H_2_O_2_ production under mitochondrial state 4 in WAT; MDA levels
[11]	Shertzer, 2010	Int J Toxicol	100 µM (water)	4.5 mg/kg/day	4 weeks	C57BL/6 mice	Female	10 weeks	NDI	4	No parameter showed an increase	4-HAE levels; MDA levels; and RCR	Weight gain; body fat; H_2_O_2_ production; mitochondrial state 3 and 4 respiration; ATP levels; thiol status
[12]	Shertzer et al., 2010	Int J Obes	100 µM (water)	4.5 mg/kg/day	10 weeks	C57BL/6 mice	Female	NA	NDI	4	O_2_ consumption and CO_2_ production; O_2_ uptake under state 4 respiration in WAT	RQ; H_2_O_2_ production under state 4 in WAT; 4-HAE; O_2_ uptake and H_2_O_2_ production using NADPH, also H_2_O_2_ production adding DPI	Body weight gain; body fat; fasting blood glucose levels; glucose tolerance; insulin levels; HOMA-IR; O2 uptake using NADPH + DPI as substrate
									HFD	4	Glucose tolerance; O_2_ consumption and CO_2_ production; O_2_ uptake under state 4 respiration in WAT	Weight gain; body fat; RQ; insulin levels; HOMA-IR; 4-HAE; O_2_ uptake and H_2_O_2_ production using NADPH, and H_2_O_2_ production using DPI	Fasting blood glucose levels; H_2_O_2_ production under mitochondrial state 4 respiration in WAT; O2 uptake using NADPH + DPI as substrate
[2]	Martin-Montalvo et al., 2016	NPJ Aging Mech Dis	35 mg/kg of chow	2.7 mg/kg/day	Longevity study	C57BL/6 mice	Male	113 weeks	NDI	16	No parameter showed an increase	No parameter showed a decrease	Lifespan; body weight trajectories
			100 µM (water)	4.5 mg/kg/day	10 weeks	C57BL/6 mice	Female	6–12 weeks	NDI	4	Cyb5r3 levels in liver and skeletal muscle	No parameter showed a decrease	Cyb5r3 levels in kidney
[3]	Watanabe et al., 2023	Sci Transl Med	100 µM (water)	4.5 mg/kg/day	4 weeks	C57BL/6 mice	NA	NA	NDI	6	No parameter showed an increase	No parameter showed a decrease	Body weight
[4]	Watanabe et al., 2024	PLoS ONE	100 µM (water)	4.5 mg/kg/day	6 weeks	C57BL/6 mice	NA	8–12 weeks	NDI	6	Insulin levels	No parameter showed a decrease	Insulin levels during glucose tolerance test; blood glucose levels; blood glucose levels under glucose tolerance test; Cyb5r3 levels in islets from pancreas

“Given dose” column specifies the concentration of THII administered in water, chow, or through gavage, while the actual intake, expressed as mg of compound per kg of body weight per day, refers to the effective dose of the compound consumed by the animals. “Time” represents the duration of the intervention with THII and the period of exposure to the specified diet mentioned. “Animal model” and “Sex” denote the rodent type, strain and sex of the animals studied. “Age” indicates the age of the animals in weeks at the beginning of the intervention. “Diet” shows whether results were reported for animals following a normal diet (NDI), a high-fat diet (HFD), or both. “Number of animals” (N. of animals) and “Results” (for increased, decreased or unchanged markers) are categorized based on animals following NDI or HFD. “NA” indicates data that were not available. *Cyb5r3* cytochrome b5 reductase 3, *DPI* diphenyleneiodonium, *EROD* ethoxyresorufin O-deethylase, *GSH-Px* glutathione peroxidase, *GSSG-Red* glutathione reductase, *GST* glutathione S-transferase, *HOMA-IR* homeostatic model assessment for insulin resistance, *MDA* malondialdehyde, *NDMA* N-nitrosodimethylamine, *Quinone reductase*, NAD(P)H:Quinone oxidoreductase, *RQ* respiratory quotient, *RCR* respiratory control ratio, *SOD* superoxide dismutase, *UDPGT* UDP-glucuronosyl transferase, *WAT* white adipose tissue, *4-HAE* 4-hydroxyalkenals

Thus, six out of the seven studies included C57BL/6 mice. In these six studies THII was administered either diluted in water at a concentration of 100 µM or supplemented in the diet at a concentration of 35 mg/kg of chow. These delivery protocols were designated to ensure an effective THII intake of 4.5 or 2.7 mg/kg of mouse body weight per day, respectively. The remaining study included Wistar rats, and THII was administered via gavage at a dose of 50 mg/kg of body weight for 10 days. Although not all details were reported, most studies that specified the sex and age of the animals enrolled female mice aged 6 to 20 weeks. The study that included a group for a longevity experiment enrolled 113-week-old male mice, while the study involving rats included male animals. Regarding the experimental conditions, results from animals on both NDI and HFD were included, with 4, 6, 8 or 16 animals per group, depending on the study and the specific experimental conditions tested. The results showed markers that either increased, decreased, or remained unchanged under the administration of THII. The qualitative analysis of the selected studies, considering the experimental conditions examined, shows that THII generally exerts a significant impact on key metabolic parameters in mice, while only minimal changes were observed in rats. In rats, the study primarily focused on levels of key liver enzymes linked to detoxification pathways. In contrast, in mice, where more outcomes were reported, the main results included parameters related to body composition, glucose homeostasis, metabolic respiration, mitochondrial respiration, oxygen uptake, ROS production, and lipid peroxidation. However, not all markers exhibited consistent pattern of change across the studies and experimental conditions. To tackle this issue, our quantitative study focused on analyzing the various metabolic markers consistently reported across the different identified studies.

Assessment of the selected studies using SYRCLE’s risk of bias tool revealed a potential moderate to high risk of bias in six out of the seven studies, with scores ranging from 4.20 to 6.30 out of 10. In contrast, the remaining study exhibited a low risk of bias, scoring 9.30 out of 10. SYRCLE’s tool highlighted that the primary sources of bias arose from an overall lack of randomization and blinding in the experimental approach (Suppl. Table 1). On the other hand, CAMARADES’s checklist reported a broader distribution of risk of bias. Two studies exhibited a potential low risk of bias (scores of 9 and 8, respectively), four studies showed a medium risk of bias (two scoring 6 and two scoring 5), and the remaining study displayed a high risk of bias (score of 4). This medium to high potential risk of bias observed in these studies was associated not only with the absence of randomization and blinding, as noted previously, but also with the lack of sample size calculation methods. Despite these concerns, both risk of bias analysis approaches indicated that all the studies were peer-reviewed, provided detailed experimental methodologies, appropriately addressed outcome data, and complied with animal welfare regulations (Suppl. Table 2).

### Effect of THII on body composition

**Fig. 1:**
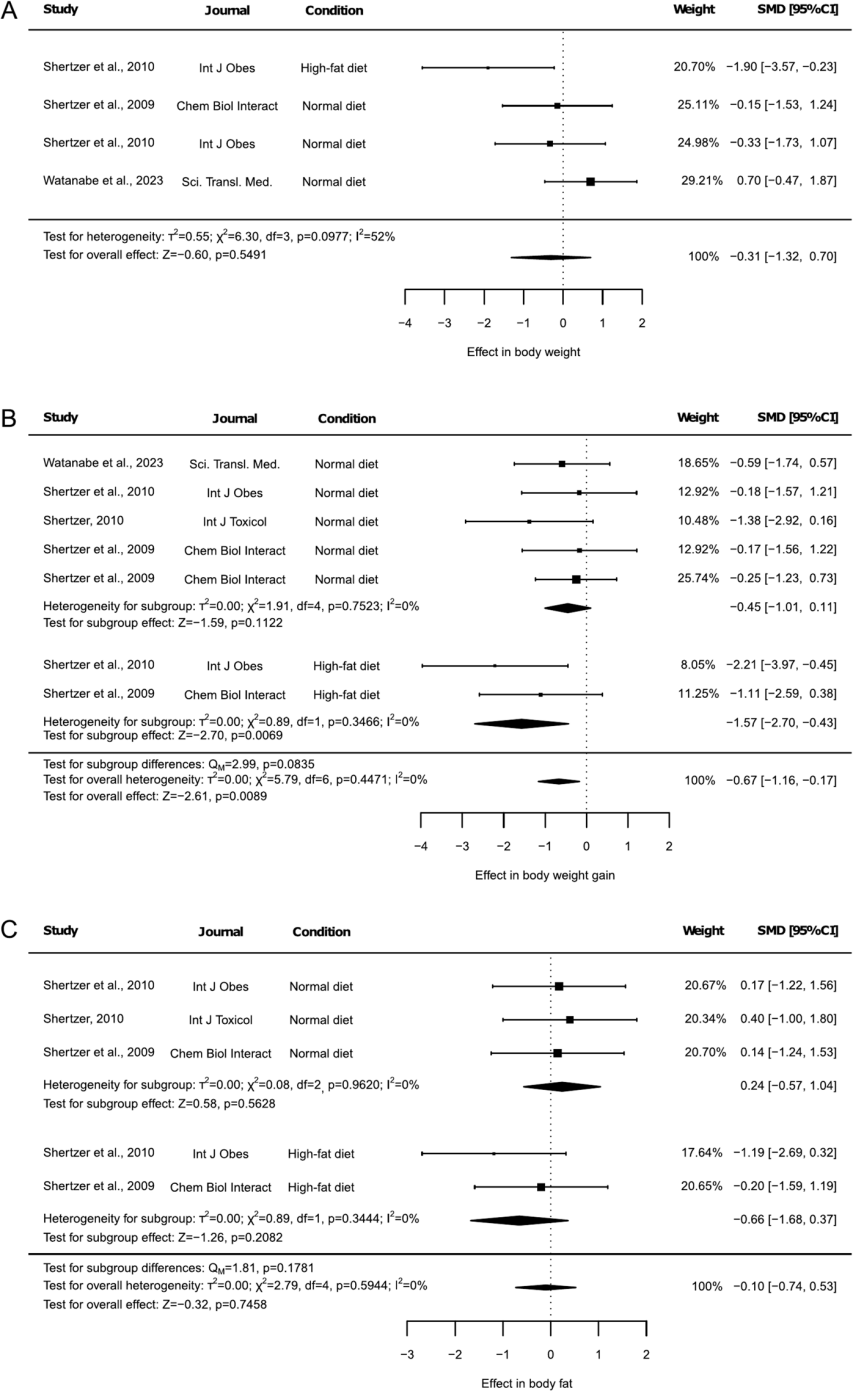
Forest plots of the effect of THII supplementation on body composition. The effects on (**A**) body weight, (**B**) body weight gain, and (**C**) body fat are shown. Each study is represented by a square with horizontal whiskers extending from either side. The position of the square indicates the SMD, the size of the square reflects the weight of the study in the meta-analysis, and the whiskers represent the 95% CI. The diamond represents the pooled effect for each subgroup or the overall effect, with its center indicating the SMD and its width corresponding to the 95% CI. Indicators of heterogeneity and subgroup effects are displayed below the corresponding subgroup. Statistical information on overall heterogeneity, overall effect, and subgroup differences are reported below all studies in each plot. SMD: standardized mean difference. 95% CI: 95% confidence interval

The effect of THII on body composition was evaluated through analyzing body weight, body weight gain and percentage of body fat (Fig. 1). The body weight was reported in 3 articles, one of them including animals under both NDI and HFD. Thus, it was reported that body weight remained unaltered under the THII supplementation (Fig. 1A, SMD: −0.31, 95% CI: −1.32 to 0.70, *p* = 0.5491). Egger's test revealed significant asymmetry in the funnel plot generated, suggesting potential publication bias (Suppl. Figure 2A, *p* = 0.0099). However, although the sensitivity analysis demonstrated that no individual study significantly influenced the overall results (Suppl. Table 3), the heterogeneity test indicated a medium degree of heterogeneity among the included studies (I^2^ = 52%, *p* = 0.0977).

The body weight gain was assessed in 4 studies, including results from both NDI and HFD. One of these studies reported results from two different groups of animals in which the effect of THII was analyzed under NDI and one group under HFD, with the two NDI groups corresponding to separate control groups from different experimental arms in Shertzer et al. (2009) (Fig. 1B). Here, THII supplementation triggered a main effect reducing body weight gain regardless of the dietary experimental condition (Fig. 1B, SMD: −0.67, 95% CI: −1.16 to −0.17, *p* = 0.0089). The subgroup analysis also revealed a reduction of body weight gain in HFD-fed animals (Fig. 1B, SMD: −1.57, 95% CI: −2.70 to −0.43, *p* = 0.0069). This effect was not observed in their control NDI-fed animals (Fig. 1B, SMD: −0.45, 95% CI: −1.01 to 0.11, *p* = 0.1122). Furthermore, the subgroup differences test revealed a tendency, with the effect of THII showing a marginal difference between animals under NDI and HFD conditions (Fig. 1B, p = 0.0835). However, no significant heterogeneity was observed (Fig. 1B, I^2^ = 0%, *p* = 0.4471) and the sensitivity test did not identify any single study that influenced the results (Suppl. Table 4). Excluding either of the two groups in which the effect of THII was evaluated under a HFD resulted in an increased p-value, although the comparison remained statistically significant (Suppl. Table 4, *p* < 0.05). Moreover, excluding any HFD group entirely eliminated the main results associated with the subgroup HFD. Additionally, Egger’s test was not significant, although a trend toward a risk of publication bias was detected (Suppl. Figure 2B, *p* = 0.0597).

The body fat mass percentage was analyzed in 3 articles, also including results from both NDI and HFD. No significant differences were observed in either the overall or subgroups tests (Fig. 1C). Similarly, no heterogeneity was detected (Fig. 1C, I^2^ = 0%, *p* = 0.5944), and the sensitivity test indicated that no single study influenced the results, although the exclusion of any HFD-related outcome led to the absence of findings for this subgroup (Suppl. Table 5). However, Egger’s test was significant, indicating a probable risk of publication bias (Suppl. Figure 2C, *p* = 0.0354).

### Effect of THII on glucose homeostasis

**Fig. 2:**
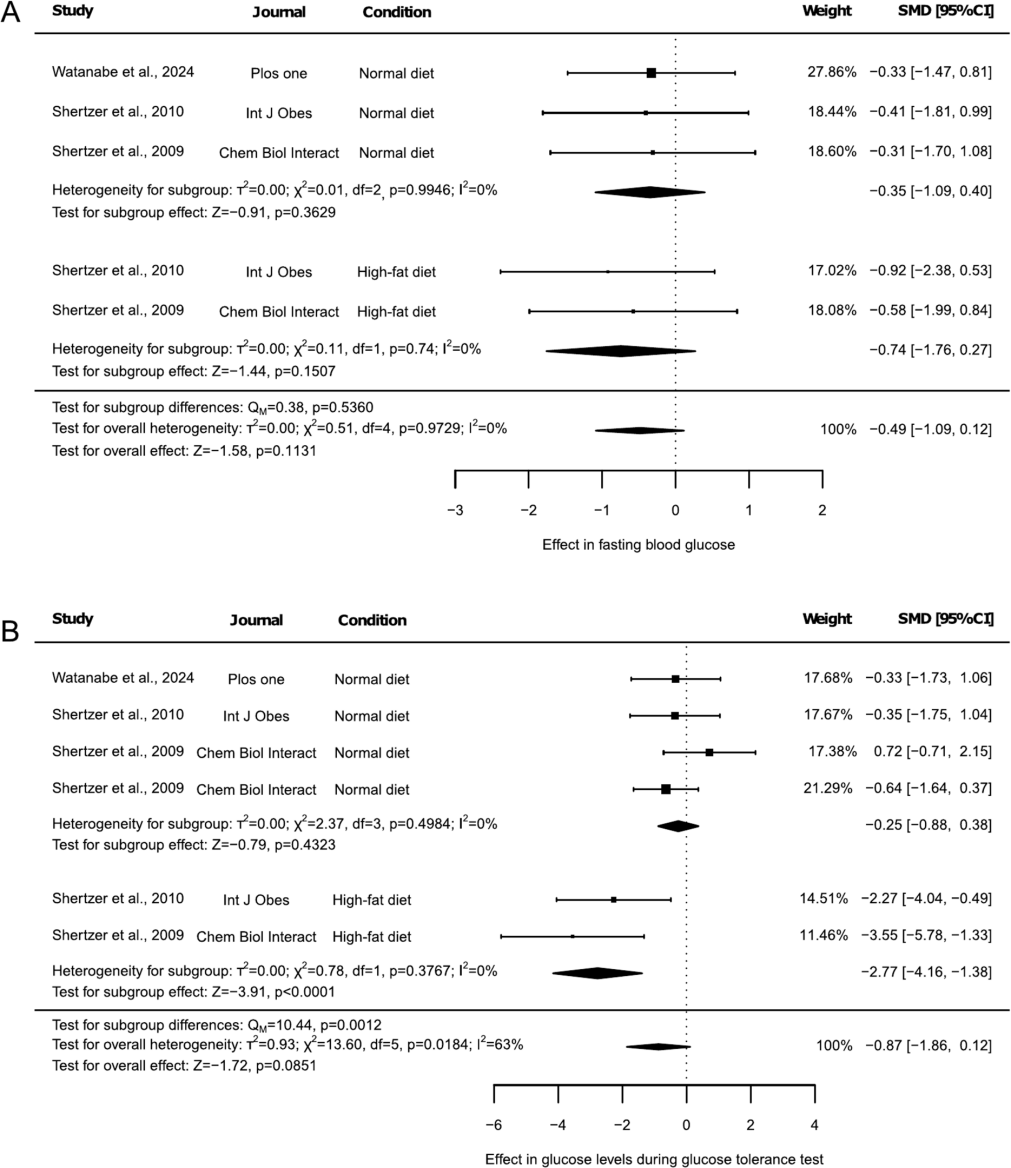
Forest plots of the effect of THII supplementation on glucose levels. The effects on (**A**) fasting blood glucose levels and (**B**) glucose levels during a glucose tolerance test are presented. Each study is represented by a square with horizontal whiskers extending from either side. The position of the square indicates the SMD, the size of the square reflects the weight of the study in the meta-analysis, and the whiskers represent the 95% CI. The diamond represents the pooled effect for each subgroup or the overall effect, with its center indicating the SMD and its width corresponding to the 95% CI. Indicators of heterogeneity and subgroup effects are displayed below the corresponding subgroup. Statistical information on overall heterogeneity, overall effect, and subgroup differences are reported below all studies in each plot. SMD: standardized mean difference. 95% CI: 95% confidence interval

**Fig. 3:**
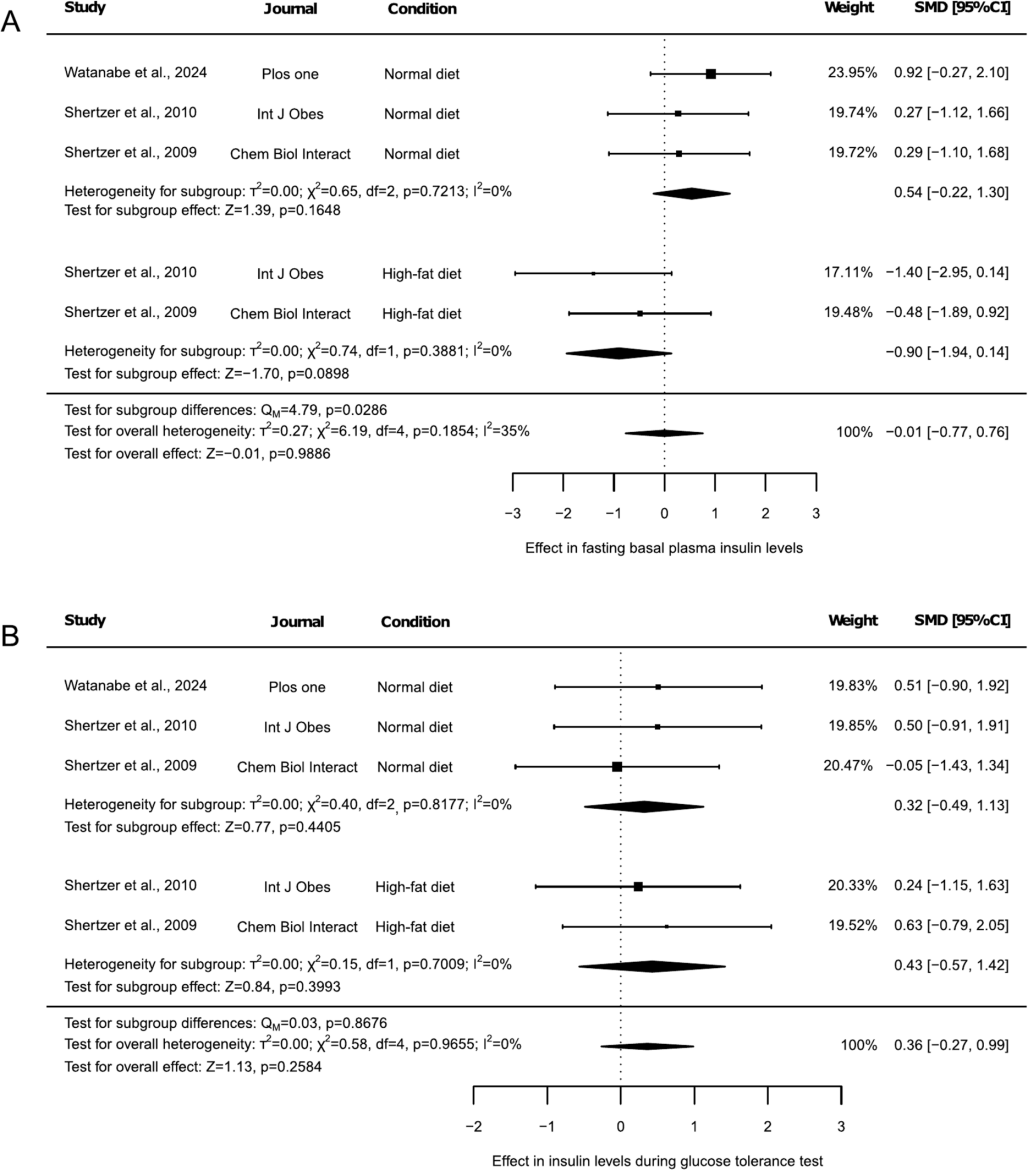
Forest plots of the effect of THII supplementation on insulin levels. The results on (**A**) fasting insulin levels and (**B**) insulin levels during a glucose tolerance test are presented. Each study is represented by a square with horizontal whiskers extending from either side. The position of the square indicates the SMD, the size of the square reflects the weight of the study in the meta-analysis, and the whiskers represent the 95% CI. The diamond represents the pooled effect for each subgroup or the overall effect, with its center indicating the SMD and its width corresponding to the 95% CI. Indicators of heterogeneity and subgroup effects are displayed below the corresponding subgroup. Statistical information on overall heterogeneity, overall effect, and subgroup differences are reported below all studies in each plot. SMD: standardized mean difference. 95% CI: 95% confidence interval

Glucose homeostasis was assessed based on blood glucose and plasma insulin levels, as well as by evaluating these parameters during a glucose tolerance test (Fig. 2 and Fig. 3). Fasting blood glucose levels were reported in 3 articles, two of which included results under both NDI and HFD conditions (Fig. 2A). Thus, glucose levels remained unchanged, both in the overall (Fig. 2A, SMD: −0.49, 95% CI: −1.09 to 0.12, *p* = 0.1131) and in the subgroup test (Fig. 2A, SMD: −0.35, 95% CI: −1.09 to 0.40, *p* = 0.3629, and SMD: −0.74, 95% CI: −1.75 to 0.27, *p* = 0.1507, for NDI and HFD, respectively), with no differences between groups (Fig. 2A, p = 0.5360). No heterogeneity (Fig. 2A, I^2^ = 0%, *p* = 0.9729), study influencing the results (Suppl. Table 6), or publication bias (Suppl. Figure 3A, *p* = 0.2996) were detected. However, the exclusion of any outcome reported under HFD resulted in the absence of findings related to this subgroup. On the other hand, glucose levels during the glucose tolerance test were assessed in 3 articles. One included only one NDI group, another study included two NDI groups and one HFD group, with the two NDI groups corresponding to separate control groups from different experimental arms of Shertzer et al. (2009), while the third included one NDI and one HFD group (Fig. 2B). Here, glucose levels during glucose tolerance test tended to decrease with THII supplementation (Fig. 2B, SMD: −0.87, 95% CI: −1.86 to 0.12, *p* = 0.0851). The subgroup test revealed that THII just decreased glucose levels during glucose tolerance test under a HFD (Fig. 2B, SMD: −2.77, 95% CI: −4.16 to −1.38, *p* < 0.0001), and these results were significantly different from those generated under NDI (Fig. 2B, *p* = 0.0012). The heterogeneity test showed an overall high heterogeneity (Fig. 2B, I^2^ = 63%, *p* = 0.0184), but no heterogeneity was observed for any subgroup (Fig. 2B, I^2^ = 0%, *p* < 0.05). The sensitivity analysis revealed that removing either of the two groups in which the effect of THII was evaluated under an HFD caused the primary observed tendency to disappear (Suppl. Table 7, *p* < 0.05), as well as any main outcome linked to HFD. In contrast, omitting the NDI group reported by Shertzer et al. (2009) that showed increased glucose levels during the tolerance test resulted in a significant decrease in the overall result (Suppl. Table 7, SMD: −1.17, 95% CI: −2.18 to −0.17, *p* = 0.0222). Moreover, exclusion of any of the other results reported by either Shertzer et al. (2009) or Shertzer et al. (2010) in the NDI subgroup also caused the tendency to disappear (Suppl. Table 7, *p* < 0.1), while removing any outcome reported under HFD resulted in the absence of finding related to his subgroup (Suppl. Table 7). Additionally, no risk of publication bias was detected (Suppl. Figure 3B, *p* = 0.1973).

Fasting basal plasma insulin levels and insulin levels during glucose tolerance test were also assessed in 3 articles, reporting two of them results from both NDI and HFD-fed animals (Fig. 3A and 3B). No overall effect was observed in fasting insulin levels (Fig. 3A, SMD: −0.01, 95% CI: −0.77 to 0.76, *p* = 0.9886). However, a decreasing trend was detected in animals under HFD (Fig. 3A, SMD: −0.90, 95% CI: −1.94 to 0.14, *p* = 0.0898), causing differences between subgroups results (Fig. 3A, *p* = 0.0286). Despite this, minor heterogeneity was observed (Fig. 3A, I^2^ = 35%, *p* = 0.1854), and no study influenced the results. However, exclusion of either of the outcomes associated with HFD resulted in a lack of results related to this subgroup (Suppl. Table 8). The risk of publication bias test reported asymmetry in the funnel plot generated (Suppl. Figure 4A, *p* = 0.0328). On the contrary, insulin levels during glucose tolerance test remained unchanged, either in the overall (Fig. 3B, SMD: 0.36, 95% CI: −0.27 to 0.99, *p* = 0.2584) or subgroup test (Fig. 3B, SMD: 0.32, 95% CI: −0.49 to 1.13, *p* = 0.4405, under NDI; SMD: 0.43, 95% CI: −0.57 to 1.42, *p* = 0.3993, under HFD), with no difference between groups (Fig. 3B, *p* = 0.8676). No heterogeneity was detected (Fig. 3B, I^2^ = 0%, *p* = 0.9655), and no single study influenced the results, although removal of either result for HFD eliminated the possibility of conducting HFD-linked subgroup-specific comparisons (Suppl. Table 9). Additionally, a potential risk of publication bias was detected (Suppl. Figure 4B, *p* = 0.0112).

### Effect of THII on mice respiratory metabolism

**Fig. 4:**
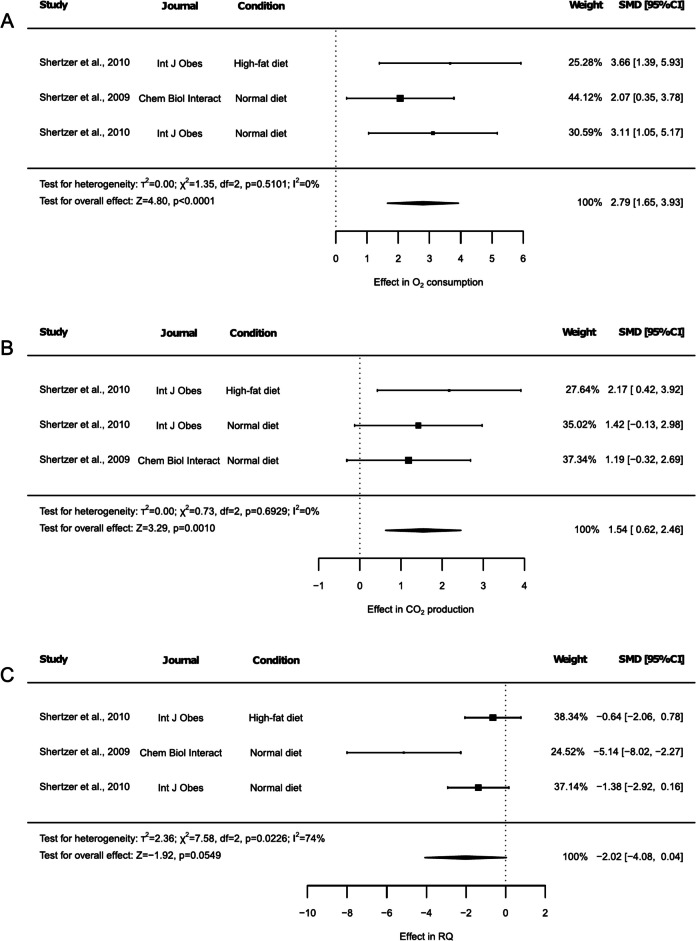
Forest plots of the effect of THII supplementation on respiratory metabolism in mice. The effects on (**A**) O_2_ consumption, (**B**) CO_2_ production, and (**C**) respiratory quotient (RQ) are displayed. Each study is represented by a square with horizontal whiskers extending from either side. The position of the square indicates the SMD, the size of the square reflects the weight of the study in the meta-analysis, and the whiskers represent the 95% CI. The diamond represents the pooled effect for the overall effect, with its center indicating the SMD and its width corresponding to the 95% CI. Statistical information on overall heterogeneity and overall effect are reported below all studies in each plot. SMD: standardized mean difference. 95% CI: 95% confidence interval

Mice respiratory metabolism parameters O_2_ consumption, CO_2_ production, and respiratory quotient (RQ) were reported in 2 studies, one including animals under both NDI and HFD (Fig. 4). Both O_2_ consumption and CO_2_ production increased due to THII (SMD: 2.79, 95% CI: 1.65 to 3.93, *p* < 0.0001, and SMD: 1.54, 95% CI: 0.62 to 2.46, p 0.0010, Fig. 4A and 4B respectively), while RQ tended to decrease (Fig. 4C, SMD: −2.02, 95% CI: −4.08 to 0.04, *p* = 0.0549).

No heterogeneity was observed in both O_2_ consumption (Fig. 4A, I^2^ = 0%, *p* = 0.5101) and CO_2_ production (Fig. 4B, I^2^ = 0%, *p* = 0.6929), although a high heterogeneity was detected for RQ (Fig. 4C, I^2^ = 74%, *p* = 0.0226). Sensitivity test revealed that none of the studies influenced the results for O_2_ consumption (Suppl. Table 10) and CO_2_ production outcomes (Suppl. Table 11), while omitting either of the two groups reported by Shertzer et al. (2010) that included RQ data resulted in the loss of the observed tendency (Suppl. Table 12, *p* < 0.1). The risk of publication bias test was significant for both O_2_ consumption (Suppl. Figure 5A, *p* = 0.0202) and CO_2_ production (Suppl. Figure 5B, *p* = 0.0301), and a tendency was observed for RQ (Suppl. Figure 5C, *p* = 0.0813).

### Effect of THII on mitochondrial respiration, O_2_ uptake, and ROS production

**Fig. 5:**
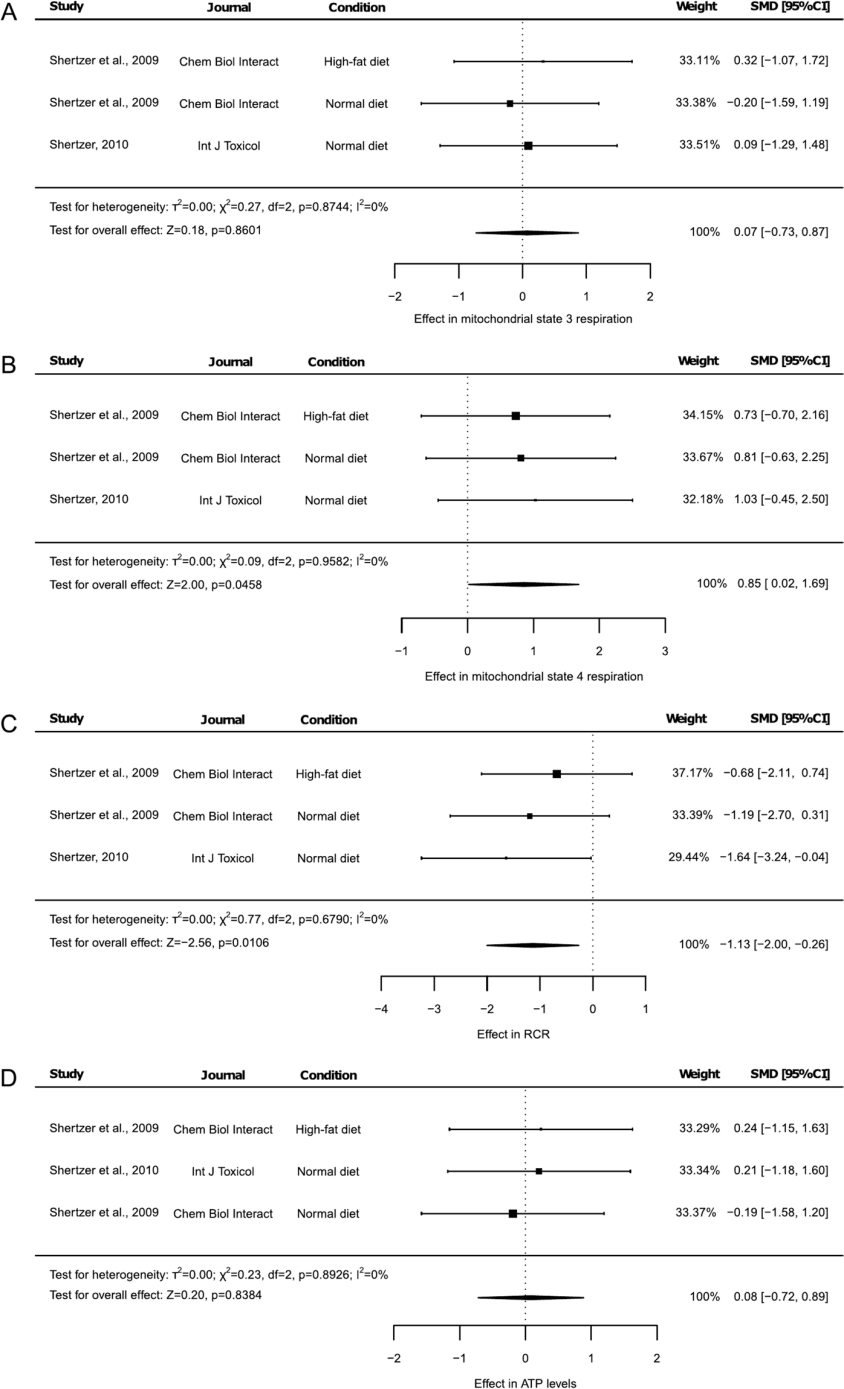
Forest plots of the effect of THII supplementation on liver mitochondrial respiration. The effects on mitochondrial (**A**) state 3 respiration, (**B**) state 4 respiration, (**C**) respiratory control ratio (RCR), and (**D**) ATP levels are presented. Each study is represented by a square with horizontal whiskers extending from either side. The position of the square indicates the SMD, the size of the square reflects the weight of the study in the meta-analysis, and the whiskers represent the 95% CI. The diamond represents the pooled effect for the overall effect, with its center indicating the SMD and its width corresponding to the 95% CI. Statistical information on overall heterogeneity and overall effect are reported below all studies in each plot. SMD: standardized mean difference. 95% CI: 95% confidence interval

Mitochondrial state 3 respiration from liver mitochondria was reported in 2 studies, one of them including animals under NDI and animals under HFD (Fig. 5A). This parameter remained unchanged under THII supplementation (Fig. 5A, SMD: 0.07, 95% CI: −0.73 to 0.87, *p* = 0.8601). These articles also assessed the mitochondrial state 4 respiration (Fig. 5B), and respiratory control ratio (RCR) under the same experimental conditions (Fig. 5C), as well as ATP levels (Fig. 5D). Mitochondrial state 4 respiration increased with THII (Fig. 5B, SMD: 0.85, 95% CI: 0.02 to 1.69, *p* = 0.0458). On the contrary, RCR decreased (Fig. 5C, SMD: −1.13, 95% CI: −2.00 to −0.26, *p* = 0.0106). Meanwhile, ATP levels remained unchanged (Fig. 5D, SMD: 0.084, 95% CI: −0.71 to 0.88, *p* = 0.8859). Here, the heterogeneity test revealed no heterogeneousness across the different results (Fig. 5, I^2^ = 0%, *p* < 0.05). Furthermore, the sensitivity test indicated that no single study influenced the results of mitochondrial state 3 respiration (Suppl. Table 13) or ATP levels (Suppl. Table 16). Regarding mitochondrial state 4 respiration, the exclusion of data reported by Shertzer (2010) increased p-value, making it non-statistically significant (Suppl. Table 14, SMD: 0.77, 95% CI: −0.25 to 1.79, *p* = 0.1376), while the omission of any of the other two groups reported by Shertzer et al. (2009) increased the p-value to indicate a trend (Suppl. Table 14, *p* < 0.1). Sensitivity test of RCR outcomes indicated that only the exclusion of data from Shertzer (2010) altered the statistical significance, increasing p-value to indicate a tendency (Suppl. Table 15, SMD: −0.92, 95% CI: −1.96 to 0.11, *p* = 0.0803). The risk of publication bias was not significant for the mitochondrial state 3 respiration and ATP levels (Suppl. Figure 6A, *p* = 0.5756 and Suppl. Figure 6D, *p* = 0.4274, respectively), while a potential risk of bias was detected for mitochondrial state 4 respiration (Suppl. Figure 6B, *p* = 0.0207), and a tendency was observed in reported mitochondrial RCR data (Suppl. Figure 6C, *p* = 0.0651).

**Fig. 6:**
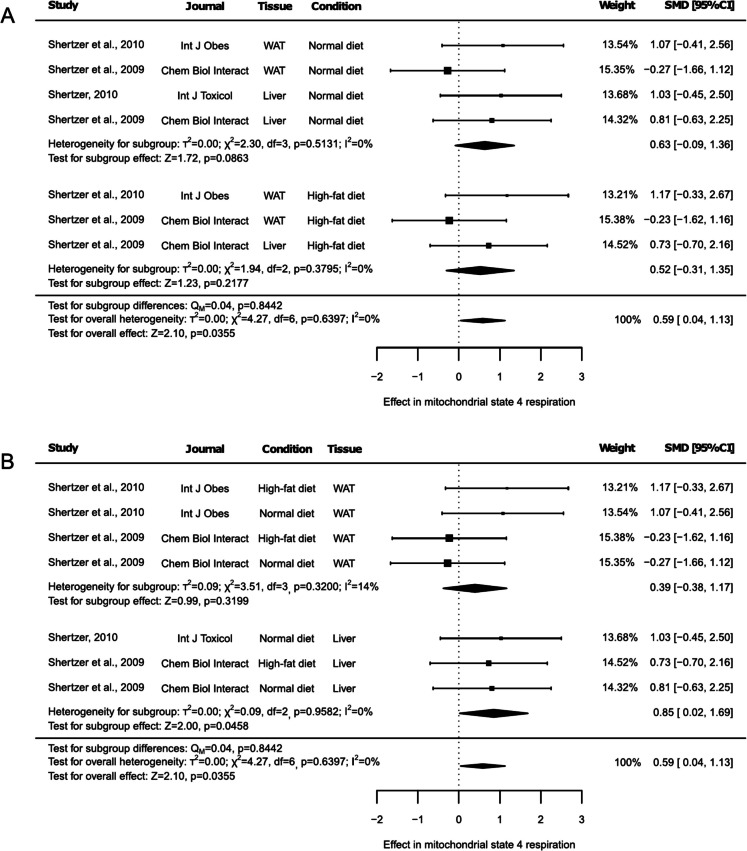
Forest plots of the effect of THII supplementation on mitochondrial state 4 respiration. The effects on mitochondrial state 4 reparation, stratified by (**A**) diet or (**B**) tissue, are showed. Each study is represented by a square with horizontal whiskers extending from either side. The position of the square indicates the SMD, the size of the square reflects the weight of the study in the meta-analysis, and the whiskers represent the 95% CI. The diamond represents the pooled effect for each subgroup or the overall effect, with its center indicating the SMD and its width corresponding to the 95% CI. Indicators of heterogeneity and subgroup effects are displayed below the corresponding subgroup. Statistical information on overall heterogeneity, overall effect, and subgroup differences are reported below all studies in each plot. SMD: standardized mean difference. 95% CI: 95% confidence interval. WAT: white adipose tissue

Beyond the outcomes described above, these articles also reported results assessing mitochondrial state 4 respiration in white adipose tissue (WAT). When considered together all these outcomes analyzing the effect of THII on mitochondrial state 4 respiration using succinate as substrate, 7 different groups were obtained, with both NDI and HFD-fed animal and with results from liver and WAT (Fig. 6A and 6B). According to the previously reported results, test for overall effect showed an increase of mitochondrial state 4 respiration under THII supplementation (Fig. 6A and Fig. 6B, SMD: 0.59, 95% CI: 0.04 to 1.13, *p* = 0.0355). Nevertheless, when dietary condition was used to generate the subgroups (Fig. 6A), the test for subgroup effect only showed a tendency in animals under an NDI (Fig. 6A, SMD: 0.63, 95% CI: −0.09 to 1.36, *p* = 0.0863). On the other hand, when tissue was used to create subgroups (Fig. 6B), the effect of THII on mitochondrial state 4 respiration was only significant for liver results (Fig. 6B, SMD: 0.85, 95% CI: 0.02 to 1.69, *p* = 0.0458). No heterogeneity was observed (I^2^ = 0%, *p* < 0.05), or only a slight but not significant heterogeneity in the WAT subgroup (Fig. 6B, I^2^ = 14%, *p* = 0.3200). Sensitivity analysis revealed that excluding any of the mitochondrial state 4 respiration data reported by Shertzer et al. (2010), Shertzer (2010), or Shertzer et al. (2009) for liver led to an increased *p*-value, indicating a trend in the overall effect (Suppl. Table 17 and Suppl. Table 18, *p* < 0.1). In the NDI subgroup, exclusion of data from Shertzer et al. (2010), Shertzer (2010), and Shertzer et al. (2009) data for liver increased the p-value, making the result non-statistically significant (Suppl. Table 17, *p* < 0.05). However, excluding only the WAT data from Shertzer et al. (2009) reduced the p-value, making the result statistically significant (Suppl. Table 17, SMD: 0.97, 95% CI: 0.12 to 1.81, *p* = 0.0251). Similarly, excluding the WAT data under HFD from Shertzer et al. (2009) resulted in a trend toward increased mitochondrial state 4 respiration with THII treatment under HFD (Suppl. Table 17, SMD: 0.94, 95% CI: −0.09 to 1.98, *p* = 0.0748). For the liver subgroup, the exclusion of any study increased the p-value. Specifically, exclusion of Shertzer et al. (2009) data resulted in a trend (Suppl. Table 18, *p* < 0.1), while exclusion of data from Shertzer (2010) made the observed change non-statistically significant (Suppl. Table 18, SMD: 0.77, 95% CI: −0.25 to 1.79, *p* = 0.1376). Additionally, a probable risk of publication bias was detected (Suppl. Figure 7A, *p* = 0.0011).

**Fig. 7:**
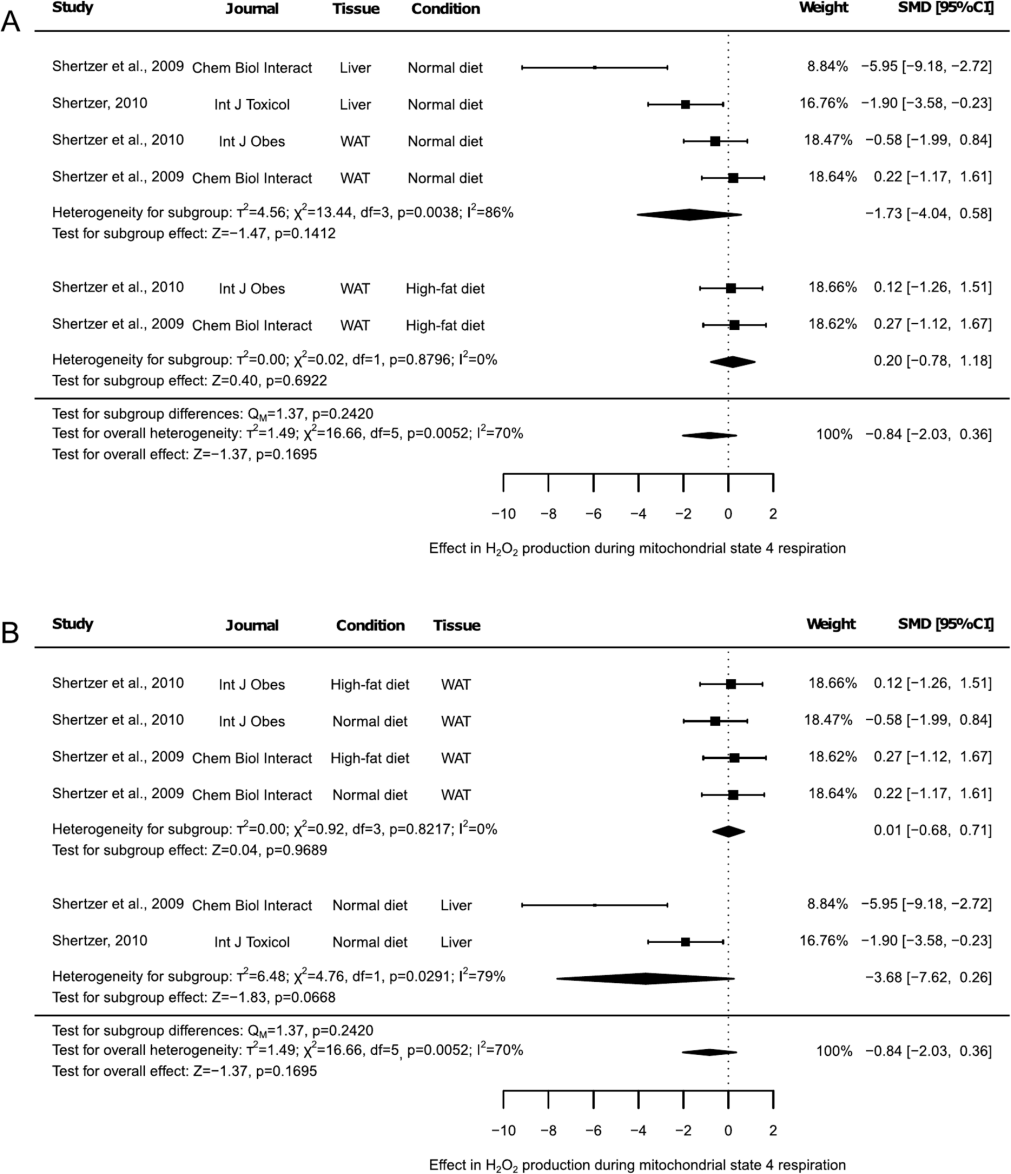
Forest plots of the effect of THII supplementation on H_2_O_2_ production during mitochondrial state 4 respiration. The effects on H_2_O_2_ production during mitochondrial state 4 respiration, stratified by (**A**) diet or (**B**) tissue, are displayed. Each study is represented by a square with horizontal whiskers extending from either side. The position of the square indicates the SMD, the size of the square reflects the weight of the study in the meta-analysis, and the whiskers represent the 95% CI. The diamond represents the pooled effect for each subgroup or the overall effect, with its center indicating the SMD and its width corresponding to the 95% CI. Indicators of heterogeneity and subgroup effects are displayed below the corresponding subgroup. Statistical information on overall heterogeneity, overall effect, and subgroup differences are reported below all studies in each plot. SMD: standardized mean difference. 95% CI: 95% confidence interval. WAT: white adipose tissue

Similarly, H_2_O_2_ production under mitochondrial state 4 respiration was reported in the same studies and experimental conditions (Fig. 7A and B). H_2_O_2_ production showed no significant changes, neither in the main THII effect (Fig. 7A and Fig. 7B, SMD: −0.84, 95% CI: −2.03 to 0.36, *p* = 0.1695) nor within individual subgroups stratified by diet. Specifically, the subgroup analysis revealed no significant differences for NDI (Fig. 7A, SMD: −1.73, 95% CI: −4.04 to 0.58, *p* = 0.1412) or HFD (Fig. 7A, SMD: 0.20, 95% CI: −0.78 to 1.18, *p* = 0.6922). However, when results were stratified by the corresponding tissue, a trend toward reduced H_2_O_2_ production was observed in the liver samples (Fig. 7B, SMD: −3.68, 95% CI: −7.62 to 0.26, *p* = 0.0668), a pattern not seen in WAT (Fig. 7B, SMD: 0.01, 95% CI: −0.68 to 0.71, *p* = 0.9689). Using the corresponding test, an overall high heterogeneity was detected (Fig. 7A and Fig. 7B, I^2^ = 70%, *p* = 0.0052), although this percentage varied between subgroups. No heterogeneity was observed in the HFD or WAT subgroup (I^2^ = 0%, *p* < 0.05), and a high and significant heterogeneity (*p* < 0.05) was observed in the NDI and liver subgroup (I^2^ = 86%, and I^2^ = 79%, respectively). The sensitivity test identified that omitting the outcome reported by Shertzer et al. (2009) for WAT under NDI conditions altered the results, indicating a trend toward reduced H₂O₂ production with THII supplementation in the NDI subgroup (Suppl. Table 19, SMD: −2.50, 95% CI: −5.38 to 0.37, *p* = 0.0880). Additionally, the exclusion of either of the two outcomes reported in the HFD subgroup made it not possible to generate HFD-associated results (Suppl. Table 19). Similarly, the exclusion of either of the two liver data results made it not possible to obtain liver-associated outcomes (Suppl. Table 20). The Egger’s test results reported a statistically significant asymmetry (Suppl. Figure 7B, *p* = 0.0052).

**Fig. 8:**
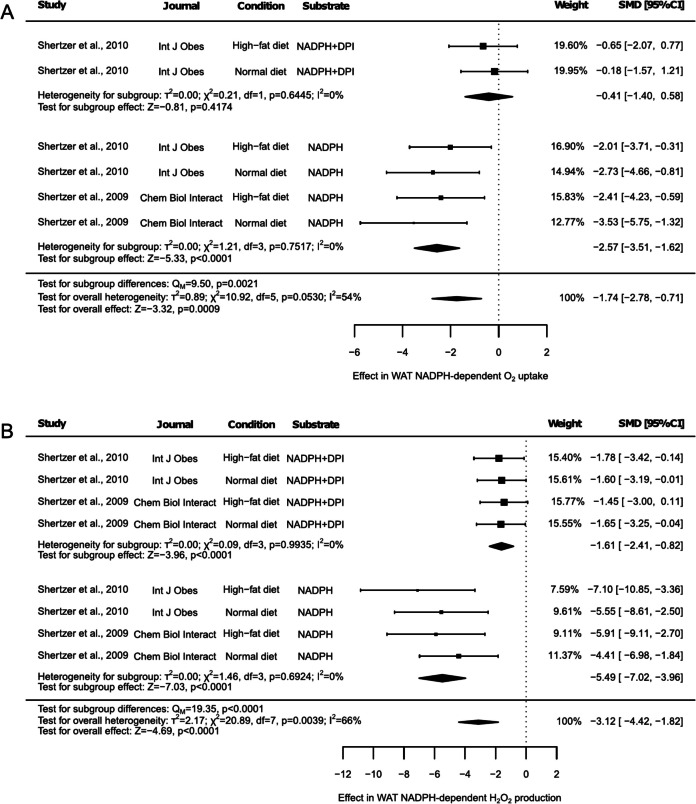
Forest plots of the effect of THII supplementation on NADPH-dependent O_2_ uptake and H_2_O_2_ production in white adipose tissue. The effects on (**A**) NADPH-dependent O_2_ uptake and (**B**) H_2_O_2_ production, with or without the inhibitor diphenyleneiodonium (DPI), are presented. Each study is represented by a square with horizontal whiskers extending from either side. The position of the square indicates the SMD, the size of the square reflects the weight of the study in the meta-analysis, and the whiskers represent the 95% CI. The diamond represents the pooled effect for each subgroup or the overall effect, with its center indicating the SMD and its width corresponding to the 95% CI. Indicators of heterogeneity and subgroup effects are displayed below the corresponding subgroup. Statistical information on overall heterogeneity, overall effect, and subgroup differences are reported below all studies in each plot. SMD: standardized mean difference. 95% CI: 95% confidence interval

O_2_ uptake using NADPH as substrate was also evaluated in WAT, both in the presence and absence of the NADPH oxidase inhibitor diphenyleneiodonium (DPI) (Fig. 8A). Two articles reported results on O_2_ uptake. One of them included two groups analyzing NADPH as a substrate and two groups analyzing NADPH as a substrate in the presence of DPI, while the other study reported results from two groups evaluating the effect of NADPH alone, without the presence of DPI. Thus, a main reduction in O_2_ consumption under these experimental conditions was associated with the effect of THII (Fig. 8A, SMD: −1.74, 95% CI: −2.78 to −0.71, *p* = 0.0009). However, subgroup analysis revealed that this effect was lost when DPI was employed (Fig. 8A, SMD: −0.41, 95% CI: −1.40 to 0.58, *p* = 0.4174). The reduction in O_2_ uptake was observed only when NADPH was evaluated alone (Fig. 8A, SMD: −2.57, 95% CI: −3.51 to −1.62, *p* < 0.001). The subgroups were significantly different from each other (Fig. 8A, *p* = 0.0021) and an overall medium heterogeneity was observed (Fig. 8A, I^2^ = 54%, *p* = 0.0530). Nevertheless, no heterogeneity was detected when subgroups were evaluated independently (Fig. 8A, I^2^ = 0%, *p* < 0.05). The sensitivity test identified no single study that influenced the overall result (Suppl. Table 21). However, the omission of any of the two results reported under the presence of DPI led to the loss of findings associated with this experimental condition (Suppl. Table 21). Additionally, the Egger’s test reported a potential risk of publication bias (Suppl. Figure 8A, *p* = 0.0004).

H_2_O_2_ production was also evaluated under the experimental conditions described above, using NADPH as a substrate, with or without DPI (Fig. 8B). Overall, a significant decrease in H_2_O_2_ production due to THII was observed (Fig. 8B, SMD: −3.12, 95% CI: −4.42 to −1.82, *p* < 0.0001). Similarly, this reduction in H_2_O_2_ production was noted when NADPH was tested alone (Fig. 8B, SMD: −5.49, 95% CI: −7.02 to −3.96, *p* < 0.0001) or in the presence of DPI (Fig. 8B, SMD: −1.61, 95% CI: −2.41 to −0.82, *p* < 0.0001). However, a significant difference between subgroups was detected (Fig. 8B, *p* < 0.0001). While overall high heterogeneity was observed (Fig. 8B, I^2^ = 66%, *p* = 0.0039), the heterogeneity test for subgroups indicated no heterogeneity (Fig. 8B, I^2^ = 0%, *p* < 0.05). Sensitivity analysis revealed that no single study significantly influenced the results (Suppl. Table 22), although a potential risk of publication bias was identified (Suppl. Figure 8B, *p* = 0).

### Effect of THII on lipid peroxidation

**Fig. 9:**
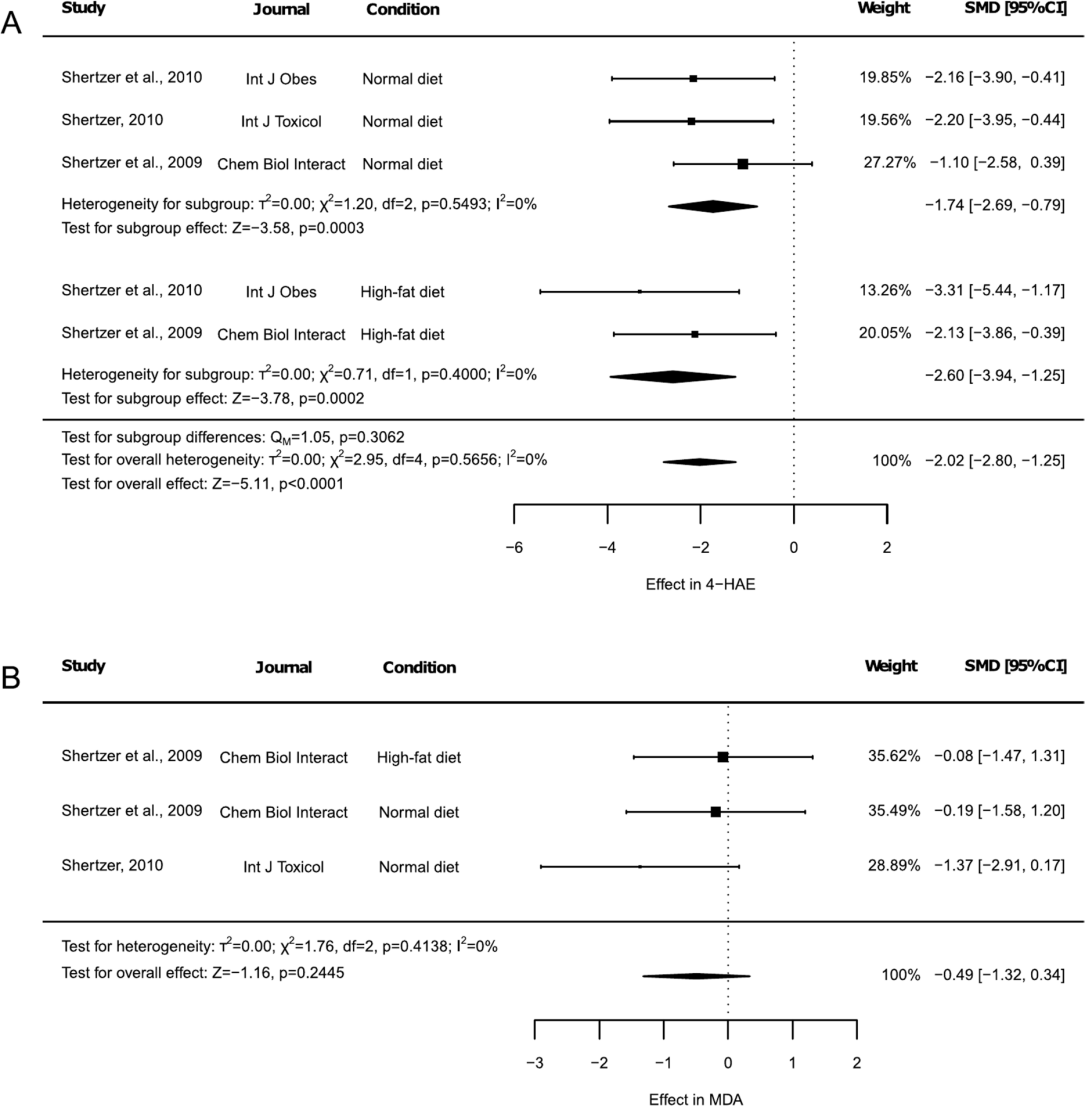
Forest plots of the effect of THII supplementation on lipid peroxidation. The effects on (**A**) 4-hydroxyalkenals (4-HAE) and (**B**) malondialdehyde (MDA) levels are shown. Each study is represented by a square with horizontal whiskers extending from either side. The position of the square indicates the SMD, the size of the square reflects the weight of the study in the meta-analysis, and the whiskers represent the 95% CI. The diamond represents the pooled effect for each subgroup or the overall effect, with its center indicating the SMD and its width corresponding to the 95% CI. Indicators of heterogeneity and subgroup effects are displayed below the corresponding subgroup. Statistical information on overall heterogeneity, overall effect, and subgroup differences are reported below all studies in each plot. SMD: standardized mean difference. 95% CI: 95% confidence interval

Lipid peroxidation was assessed by analyzing 4-hydroxyalkenals (4-HAE) and malondialdehyde (MDA) (Fig. 9). 4-HAE levels were reported in 3 studies, including both NDI and HFD groups (Fig. 9A). THII triggered an overall reduction of 4-HAE levels (Fig. 9A, SMD: −2.02, 95% CI: −2.80 to −1.25, *p* < 0.0001), being these results also independently significant under both NDI (Fig. 9A, SMD: −1.74, 95% CI: −2.69 to −0.79, *p* = 0.0003) and HFD (Fig. 9A, SMD: −2.60, 95% CI: −3.94 to −1.25, *p* = 0.0002), with no difference between subgroups (Fig. 9A, *p* = 0.3062). No heterogeneity was observed (Fig. 9A, I^2^ = 0%, *p* = 0.5656). The sensitivity analysis revealed no article that modified the results, although the exclusion of any HFD-related outcome resulted in the absence of results linked to this subgroup (Suppl. Table 23). Additionally, Egger’s test resulted statistically significant (Suppl. Figure 9A, *p* = 0.0009).

In contrast, MDA was determined in 2 articles, one including both NDI and HFD-fed animals, while the other included only NDI-fed animals (Fig. 9B), but no effect linked to THII was detected (Fig. 9B, SMD: −0.49, 95% CI: −1.32 to 0.34, *p* = 0.2445). Finally, the heterogeneity test revealed no significant variability (Fig. 9B, I^2^ = 0%, *p* = 0.4138), while sensitivity test indicated that no single article influenced the results (Suppl. Table 24), and the risk of publication bias was statistically significant (Suppl. Figure 9B, *p* = 0.0439).

## Discussion

Indenoindole and its derivatives represent a group of compounds that have been demonstrated to possess antitumoral [9, 19–21], antiapoptotic [22], chemoprotective [8, 11, 12, 23–25], and antioxidant properties [1, 8, 26–32]. Featuring a central fused ring structure composed of 6–5-5–6-membered rings [33], these compounds have been associated with multiple potential processes and pathways, suggesting various mechanisms of action when administrated. These compounds have been shown to act as free radical quenchers [26], as well as inhibitors [19] or activators [2] of certain proteins. However, their exact mechanism of action is not completely understood.

Among them, THII has gained special interest due to its promising beneficial effect in both cellular and animal models, suggesting its potential for translational applications. For example, THII inhibited lipid peroxidation initiated by carbon tetrachloride (CCl_4_) in rat liver microsomes [8], restored the reduced responsiveness of insulin secretion induced by glibenclamide (GLB) in mouse and human pancreatic islets [3], and reversed glucose-stimulated insulin secretion dysfunction displayed in Min6 cells and isolated human islets under interleukin-22 receptor subunit alpha 1 (IL-22RA1) knockdown [5]. Furthermore, in vivo administration of THII in rats protected against CCl_4_-induced hepatotoxicity and induced the expression of both antioxidant and detoxification enzymes [8]. In vivo administration of THII also reversed GLB-induced impairments in insulin secretion and glucose homeostasis impairment [3, 4]. THII has also been shown to retard chemically induced skin carcinogenesis in mice [9], to protect against 2,3,7,8-tetrachlorodibenzo-p-dioxin toxicity [11], as well as to inhibit streptozotocin-induced type I diabetes-like effects [10] and olanzapine-induced metabolic dysfunction [12]. Similarly, THII supplementation restored glucose metabolism alterations observed in transgenic IL-22RA1-deficient mice, improving glucose tolerance, increasing fasting plasma insulin levels, and reducing both fasted and fed blood glucose, while also rescued insulin levels, ATP production, and maximal mitochondrial respiration impairment triggered by IL-22RA1 deficiency in isolated islets [5].

Despite the reported results outlined above, the study of the association between THII supplementation and the promotion of health and healthspan has received limited attention, even though a potential connection appears evident in certain respects. In mice fed HFD, THII delayed diabetogenesis by improving glucose and insulin homeostasis, fat and energy metabolism, and oxidative stress-associated pathways [10, 12]. Nevertheless, the effects of THII supplementation under standard control diet, reflective of normal physiological state, or within the context of natural aging, remain largely unexplored.

To further contribute to the understanding of the effects of THII supplementation in a non-disease health context, this systematic review with meta-analysis was performed, evaluating previously published data from a novel perspective to provide new and summarized insights. Thus, our approach demonstrates that THII supplementation not only mitigates various alterations caused by pathological conditions through enhancing metabolism, optimizing mitochondrial function, and reducing oxidative stress, but also induces these beneficial effects under normal physiological conditions in the absence of disease. Based on these findings, THII supplementation does not affect body weight or fat mass but reduces body weight gain regardless of the dietary pattern followed, with this effect being particularly pronounced in HFD-fed animals. This outcome may be attributed to decreased fat absorption or accumulation, and increased energy expenditure, potentially linked to enhanced metabolism, particularly fat metabolism, and elevated thermogenesis [34]. Supporting this, increased O_2_ consumption, elevated CO_2_ production, and reduced respiratory quotient are observed in liver, consistent with a shift toward enhanced fat oxidation [35].

While fasting blood glucose and insulin levels remained unchanged, THII supplementation significantly improved glucose tolerance, with the effect being remarkably pronounced in HFD-fed animals. This suggests enhanced glucose homeostasis, a critical factor for maintaining overall metabolic health [36]. Moreover, these findings also suggest that THII may be especially effective in managing postprandial hyperglycemia, a key contributor to diabetes progression [37]. The lack of changes in fasting glucose and insulin levels implies that THII does not alter basal metabolism, potentially minimizing the risk of hypoglycemia, which is a common concern associated with some glucose-lowering interventions [38].

Furthermore, although mitochondrial state 3 respiration remains unaltered, state 4 respiration increases with THII supplementation, leading to a reduction in RCR. Such changes in mitochondrial state 4 respiration and RCR have been associated with both beneficial and detrimental effects [39]. However, in this case, the observed absence of either reduced ATP production or increased mitochondrial-dependent H_2_O_2_ production points toward a predominantly beneficial effect. Increased mitochondrial state 4 and reduced RCR, without a concomitant rise in ROS production, has been associated with improved mitochondrial capacity to metabolize substrates efficiently through electron transport chain uncoupling [40–42]. This mechanism is particularly important under conditions of nutrient excess and it may be linked to the optimization of fat metabolism and enhanced mitochondrial functionality [43]. Moreover, the impact on reducing NADPH enzymes-dependent O_2_ uptake and H_2_O_2_ production may also shed light about potential mechanisms and pathways through which THII triggers the exhibited changes, suggesting that the effects could also be mediated by modulation of NADPH oxidases activity, as evidenced by the partial reversal of these reductions upon DPI, a known unspecific inhibitor of NADPH oxidase [44]. As described previously, an overall reduction of lipid peroxidation is also observed upon THII treatments. However, the changes in both parameters assessed were not identical, suggesting that THII may reduce lipid peroxidation through a specific pathway or compensatory mechanism, as different lipid peroxidation products follow different pathways or exhibit varying reactivity [45].

Tissue- and dietary pattern-dependent effects of THII supplementation have been demonstrated. The reduction in body weight gain with no alteration in body fat percentage, supported by changes observed in energy metabolism, suggests an improved ability to metabolize the lipids present in the diet due to THII supplementation, which is particularly important in diets with a high fat content [46]. Moreover, glucose tolerance was also enhanced, although fasting glucose and insulin levels remained unchanged. All these changes may be linked to specific changes triggered by THII in peripheral tissues, especially skeletal muscle, liver, or adipose tissue. For instance, THII supplementation could lead to enhanced lipolysis in adipose tissue, improved fatty acid oxidation in skeletal muscle, and/or optimized lipid oxidation and transport in liver. Furthermore, the improved glucose tolerance without changes in fasting glucose and insulin levels likely reflects enhanced insulin sensitivity and/or glucose effectiveness in these tissues due to THII supplementation, notably in skeletal muscle, which is responsible for up to 85% of total glucose uptake [47].

The beneficial effects of THII have been linked with a mechanism involving the enzyme CYB5R3 [3, 4]. Evidence for this connection derives from studies using CYB5R3 knockout animals, where THII failed to induce the effects observed in wild-type individuals [3]. Despite these findings, the precise mechanism of action remains incompletely understood. THII is recognized as an antioxidant that acts via electron donation to free radicals [1]. Thus, a plausible mechanism might involve electron transfer from THII to CYB5R3, enhancing its enzymatic activity and promoting electron transfer to its substrates. In this context, THII would undergo oxidation and thus require subsequent reduction to maintain its function within the redox cycle. An alternative possibility is that THII would accept electrons from CYB5R3, preserving its capacity to donate electrons to free radicals and neutralizing them.

Although the mechanism is not yet fully elucidated, dietary THII supplementation has been reported to increase relative *Cyb5r3* transcript levels in the liver and skeletal muscle from female mice [2]. Interestingly, this increase was more pronounced in skeletal muscle, followed by the liver, while no significant change was observed in kidney, further supporting the potential tissue-dependent effects of THII supplementation and the tissue-specific pathways involved. CYB5R3 overexpression is a well-characterized anti-aging intervention with effects that mimic part of those triggered by calorie restriction, such as improved glucose homeostasis, reduced oxidative damage, and enhanced protection against cancer [2]. Additionally, CYB5R3 overexpression has been reported to exhibit not only tissue-specific effects but also sex-dependent outcomes [48], a situation that may also occur with THII supplementation. Moreover, CYB5R3 overexpression has been shown to maintain mitochondrial status and mitigate oxidative stress [2, 49], further supporting a mechanistic connection between THII supplementation, mitochondrial function, and redox homeostasis. Altogether, this stresses the capability of THII to promote health and accentuates its potential relevance in the field of aging-related research.

Notwithstanding the promising outcomes reported and the impact of THII on key metabolic parameters, further studies are needed to better understand its effects, first in preclinical animal models, ahead of advancing to human applications. In this regard, it is essential to have a comprehensive understanding of prior research to identify and address existing limitations, emphasize the replicability of previously reported key outcomes, and explore potential new outcomes that may arise due to the impact of the intervention. Here, several limitations and potential biases have been identified in the existing literature. One of the primary concerns is the limited number of studies available and the restricted diversity of research groups working on this topic, which may contribute to selection and publication bias. Furthermore, the findings to date are primarily based on data derived from female animals, limiting the generalizability of the results across sexes. Future studies should prioritize the inclusion of both male and female subjects to explore, in parallel, the effects of the intervention in each sex. Another aspect to consider is the tissue-dependent effect observed in the outcomes, which emphasizes the need for more investigations to determine whether the intervention exhibits differential effects across the different tissues and/or organs. The reported results are focused on a limited number of blood markers, primarily glucose and insulin levels, while a large number of body composition parameters remain unanalyzed, as only data on weight, weight gain, and fat percentage have been reported. Furthermore, metabolic studies in tissues have been restricted to muscle and liver. So, multi-tissue analysis should be conducted in future studies. Additionally, there is a notable absence of data regarding the use of alternative doses or studies that provide a robust base for the specific dosage employed in the reported experiments. Conducting dose–response studies that assess a wider range of doses, including both lower and higher concentrations than those currently reported, would provide a more comprehensive understanding of the efficacy and safety of the intervention. In vivo studies have primarily used a dose of 4.5 mg of THII/kg body weight/day, administered by diluting the compound in water, or a dose of 2.7 mg of THII/kg body weight/day, supplemented in the diet. This also raises the possibility of evaluating the effect of administering the compound at these doses through either water or diet, to assess whether the method of administration influences the outcomes. Moreover, current studies have largely focused on a limited age range and specific durations of exposure to the compound. The reported ages indicate that animals used in the studies were primarily between 6 and 12 weeks old, with intervention periods ranging from 4 to 10 weeks. Additionally, in the longevity study involving THII, compound administration began when the animals were 113 weeks old, meaning they had already reached an advanced age, surpassing two years of life. Thus, including individuals at various stages throughout their entire life cycle and longer exposure periods would help determine long-term effects and potential windows of sensitivity.

In addition, the sensitivity analysis revealed that certain studies had a significant impact on the results of specific markers. In the HFD subgroup, where the number of studies was limited, removing any single study often led to the loss of subgroup-associated effects. In contrast, the NDI subgroup was less affected by the exclusion of individual studies, likely due to the larger number of reported outcomes under this experimental condition. Parameters such as glucose tolerance, respiratory quotient, and mitochondrial state 4 respiration were particularly sensitive, with the removal of specific outcomes shifting significant results to trends, or trends to non-significance. Shertzer et al. (2009) had a pronounced impact on these parameters, especially when liver and WAT data were combined, as seen in the mitochondrial state 4 respiration results. Interestingly, excluding WAT data from this study often increased the observed effect sizes, while excluding other studies generally reduced or eliminated statistical significance, which may further support the previously described tissue-specific responses. Despite its influence, this study did not show a high risk of bias, and all included studies met the quality standards required for publication in peer-reviewed journals. Overall, although some results were sensitive to individual data entries, most results remained consistent, with low or no heterogeneity, supporting the robustness of the findings, and additional data from future studies would help to validate these results and clarify the contribution of specific studies, tissues, or conditions to the observed effects.

Finally, the main sources of bias identified through the risk of bias analysis, such as the overall lack of blinding, randomization, and appropriate methods for calculating sample size, further highlight the need for methodological improvements. Lack of blinding and randomization may introduce unintended biases in outcome assessment, potentially overestimating or underestimating the actual effects of THII. Furthermore, lack of appropriate methods for calculating sample sizes raises concerns about statistical power of reported findings. Given these issues, the discussion of the results must acknowledge these limitations and the potential impact of bias on effect sizes. Addressing these limitations will be crucial for enhancing the reliability, replicability, and overall validity of future research on this intervention, facilitating the advancement toward potential clinical trials in humans. THII has been observed to exert a significant beneficial effect on obesity, type I and type II diabetes, and metabolic dysfunction in preclinical models. In addition, our study discusses that THII also has a promising effect on overall physiological function in the context of health and physiological aging. These outcomes highlight the potential relevance of THII for human health and its possible translation into clinical applications.

Beyond these limitations, the published studies to date have provided valuable contributions, offering promising insights and establishing a solid foundation for future research. Taken together, our meta-analysis findings highlight that THII supplementation induces beneficial metabolic effects not only under high-fat diet conditions but also under standard diet in female mice, within the context of the dosage patterns, intervention durations, age ranges, and tissues analyzed and mentioned across the study. These effects include improved glucose tolerance, enhanced fat metabolism, optimized mitochondrial function, and reduced oxidative stress. Addressing the identified gaps and incorporating the suggested approaches in future investigations will be essential to contribute to fully elucidate the impact and potential of THII interventions, ultimately facilitating their translational application to clinical practice.

## Supplementary Information

Below is the link to the electronic supplementary material.

Supplementary file1 (DOCX 5.33 MB)

Supplementary file2 (DOCX 69 KB)

## Data Availability

The data and scripts utilized in this study are available from the corresponding authors upon reasonable request.
