# Supplementary material for: Effects of tetrahydroindenoindole supplementation on metabolism: A systematic review with meta-analysis of rodent-based studies
Source: GeroScience. 2025 May 5;48(1):727–53. doi: 10.1007/s11357-025-01680-z (PMC12972266; doi:10.1007/s11357-025-01680-z)
Supplement: Supplementary file 1 — Supplementary file1 (DOCX 5.33 MB) [file 11357_2025_1680_MOESM1_ESM.docx]

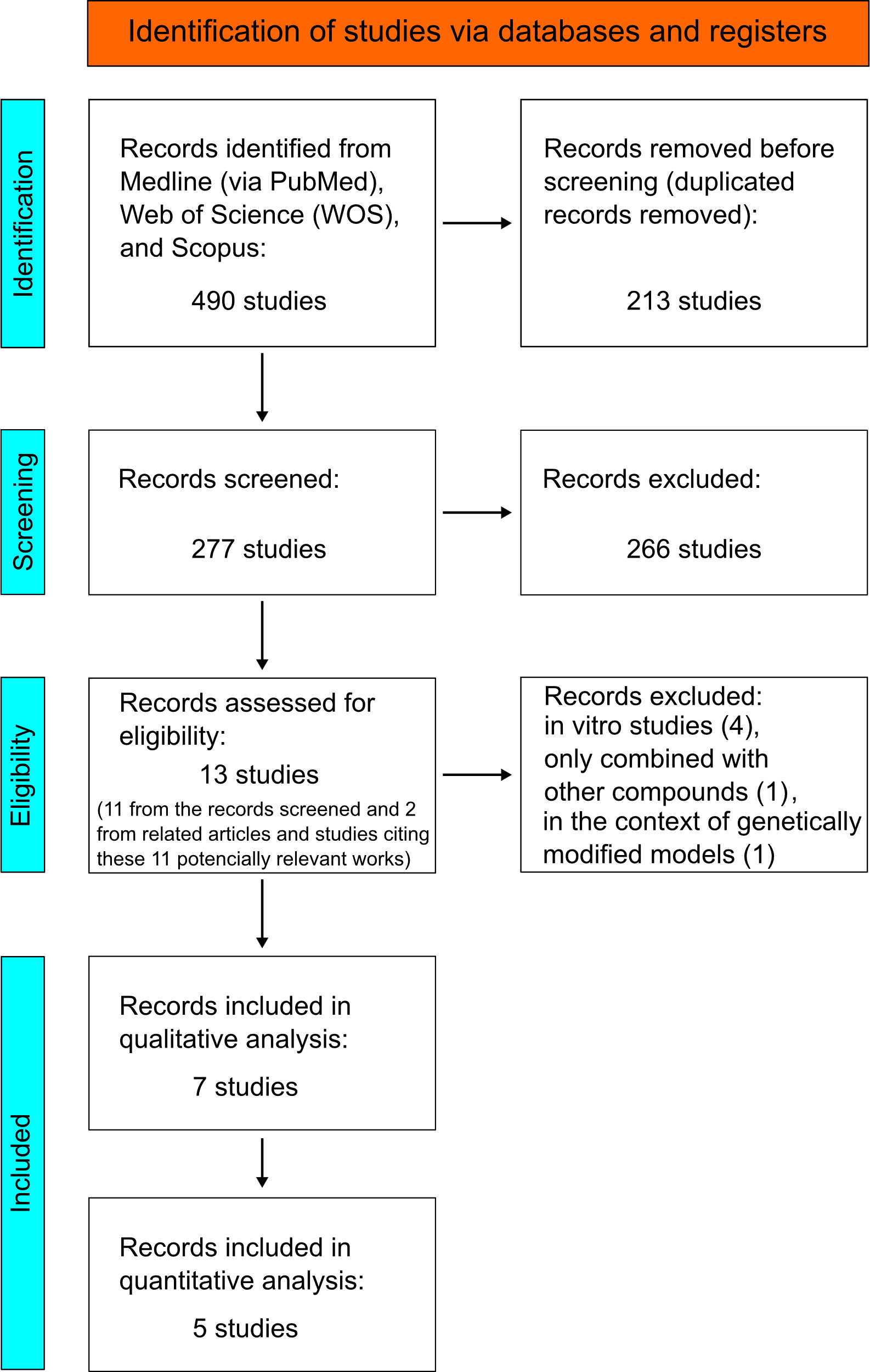


**Suppl. Fig. 1** Flowchart of the study selection process for the systematic review and meta-analysis


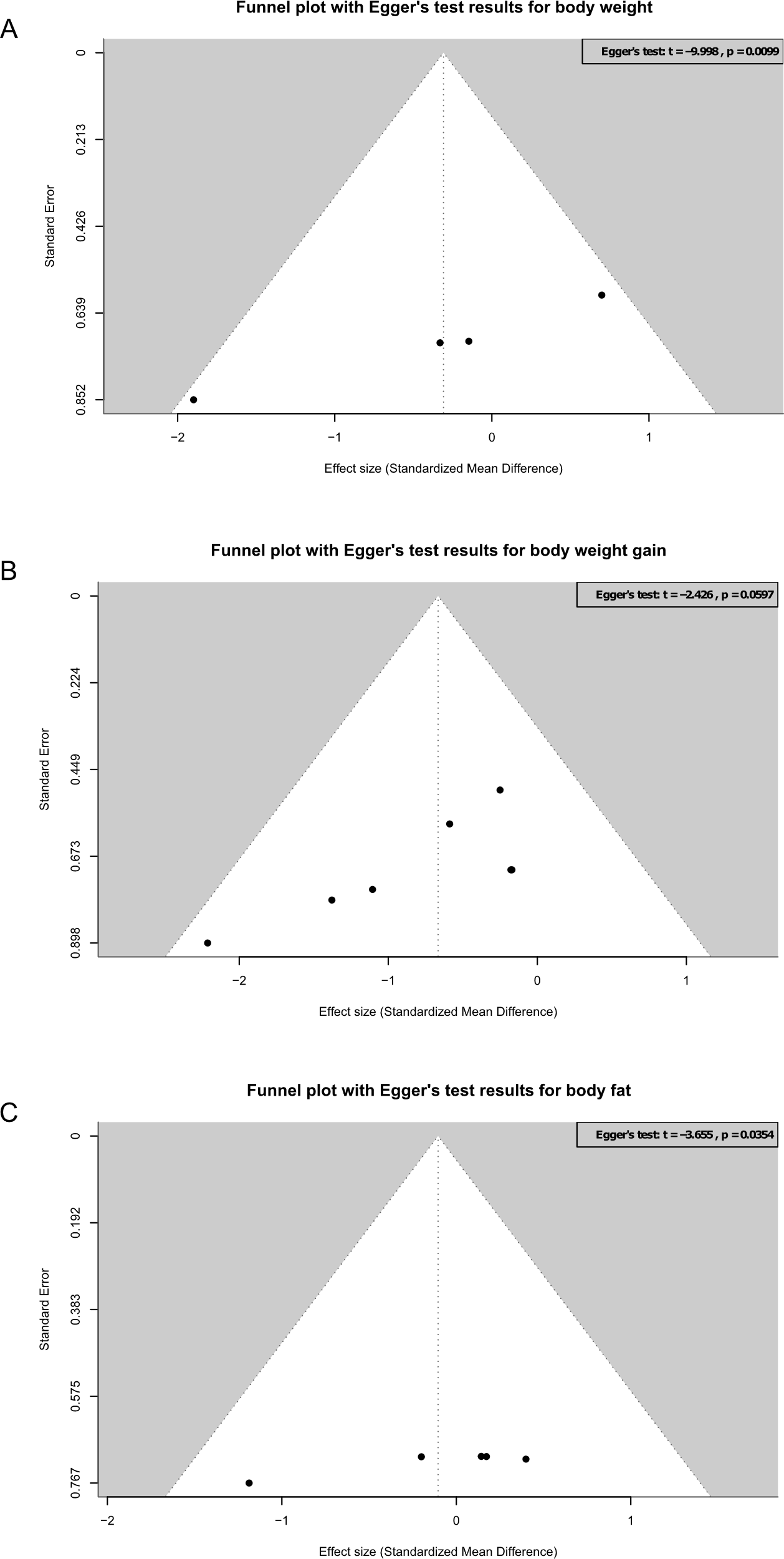


**Suppl. Fig. 2** Funnel plots for the studies included in body composition analysis. Funnel plots assessing the risk of publication bias in the studies that analyzed (A) body weight, (B) body weight gain, and (C) body fat are presented. Each plot visualizes the distribution of studies effects and includes results from Egger's test to evaluate asymmetry, indicating potential bias (p < 0.05)


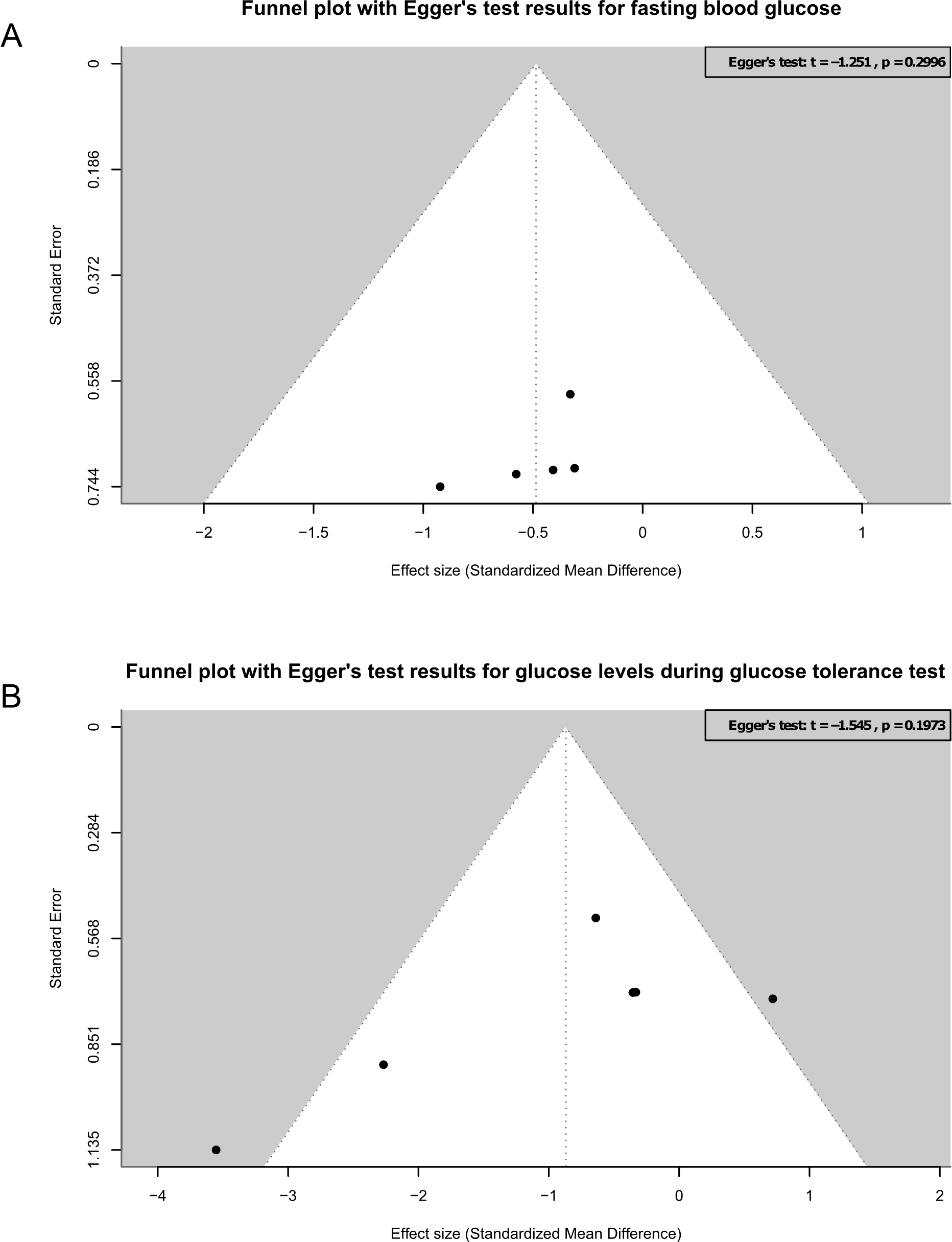


**Suppl. Fig. 3** Funnel plots for studies included in glucose levels analysis. Funnel plots assessing the risk of publication bias in the studies that analyzed (A) fasting blood glucose levels and (B) glucose levels during a glucose tolerance test are presented. Each plot visualizes the distribution of studies effects and includes results from Egger's test to evaluate asymmetry, indicating potential bias (p < 0.05)


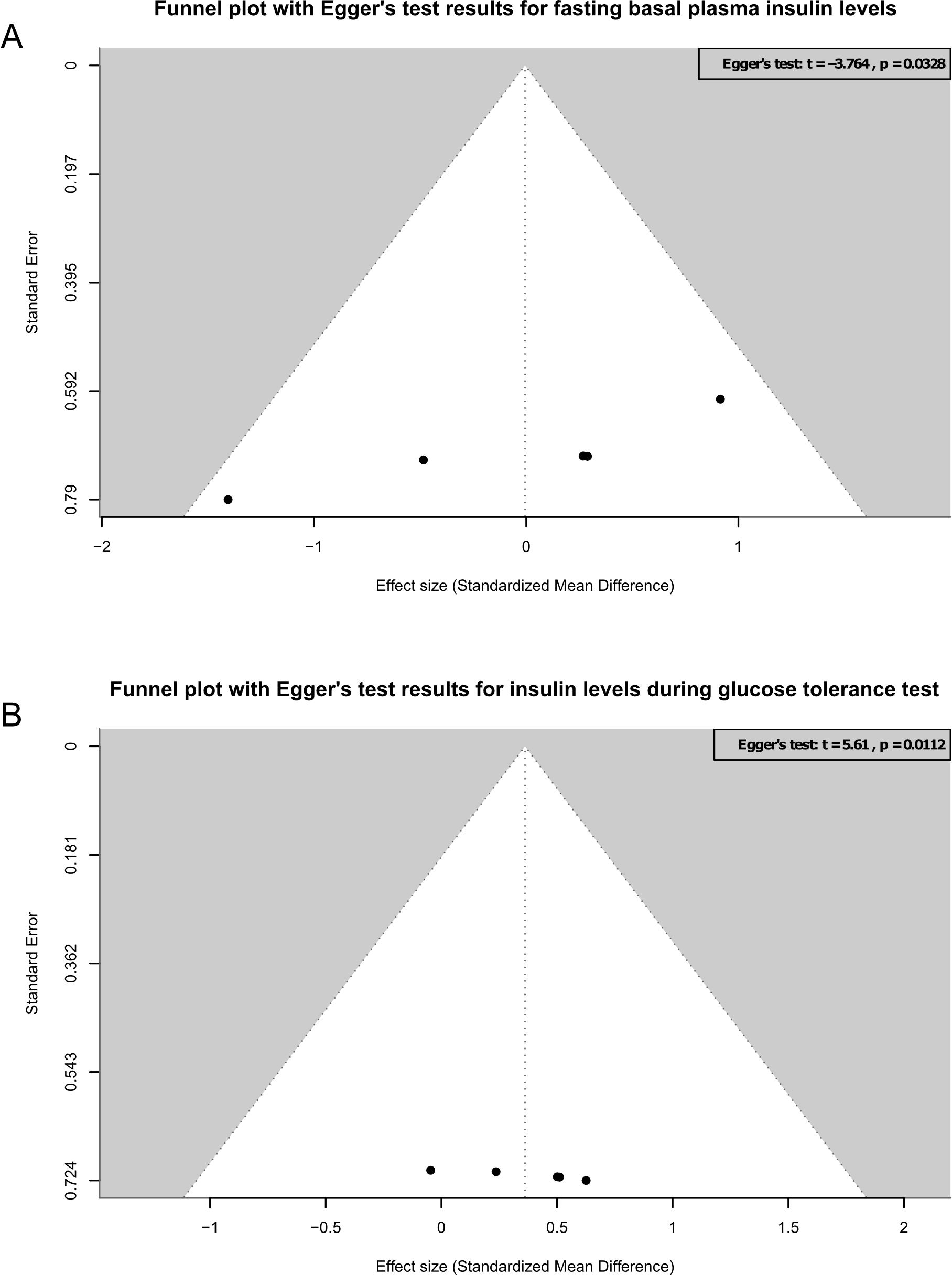


**Suppl. Fig. 4** Funnel plots for studies included in insulin levels analysis. Funnel plots assessing the risk of publication bias in the studies that analyzed (A) blood insulin levels and (B) insulin levels during a glucose tolerance test are presented. Each plot visualizes the distribution of studies effects and includes results from Egger's test to evaluate asymmetry, indicating potential bias (p < 0.05)


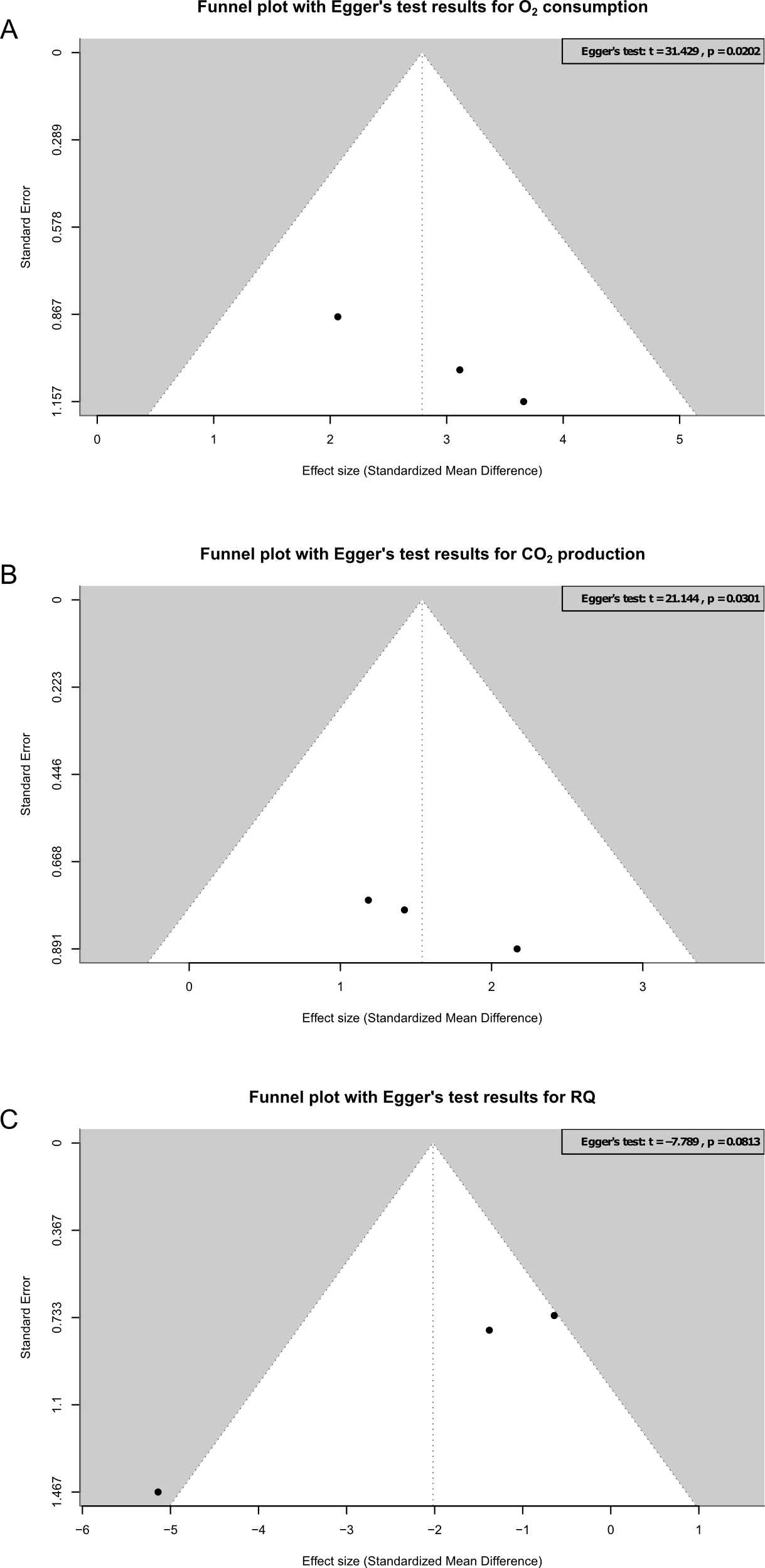


**Suppl. Fig. 5** Funnel plots for studies included in the analysis of mice respiratory metabolism. Funnel plots assessing the risk of publication bias in the studies that analyzed (A) O_2_ consumption, (B) CO_2_ production, and (C) respiratory quotient (RQ) are presented. Each plot visualizes the distribution of studies effects and includes results from Egger's test to evaluate asymmetry, indicating potential bias (p < 0.05)


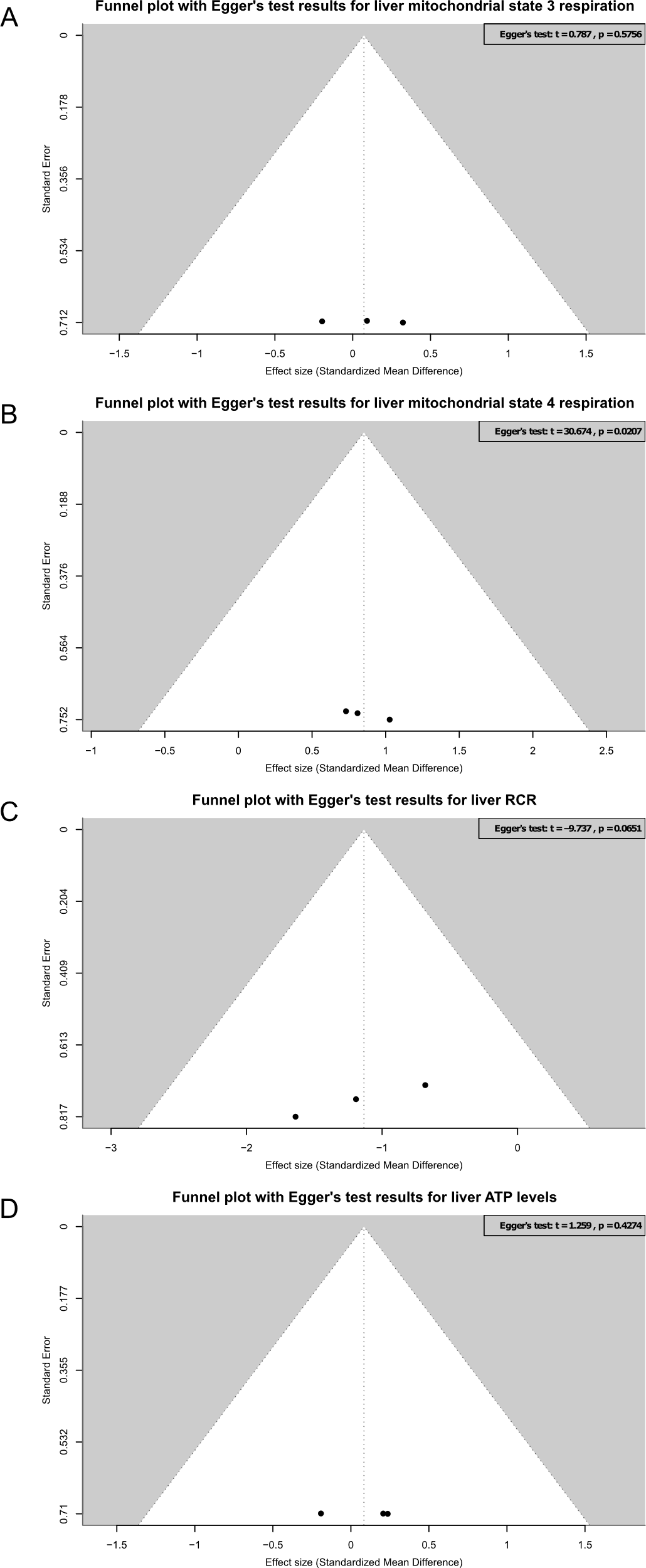


**Suppl. Fig. 6** Funnel plots for studies included in the analysis of liver mitochondrial respiration. Funnel plots assessing the risk of publication bias in the studies that analyzed mitochondrial (A) state 3 respiration, (B) state 4 respiration, (C) respiratory control ratio (RCR), and (D) ATP levels are presented. Each plot visualizes the distribution of studies effects and includes results from Egger's test to evaluate asymmetry, indicating potential bias (p < 0.05)


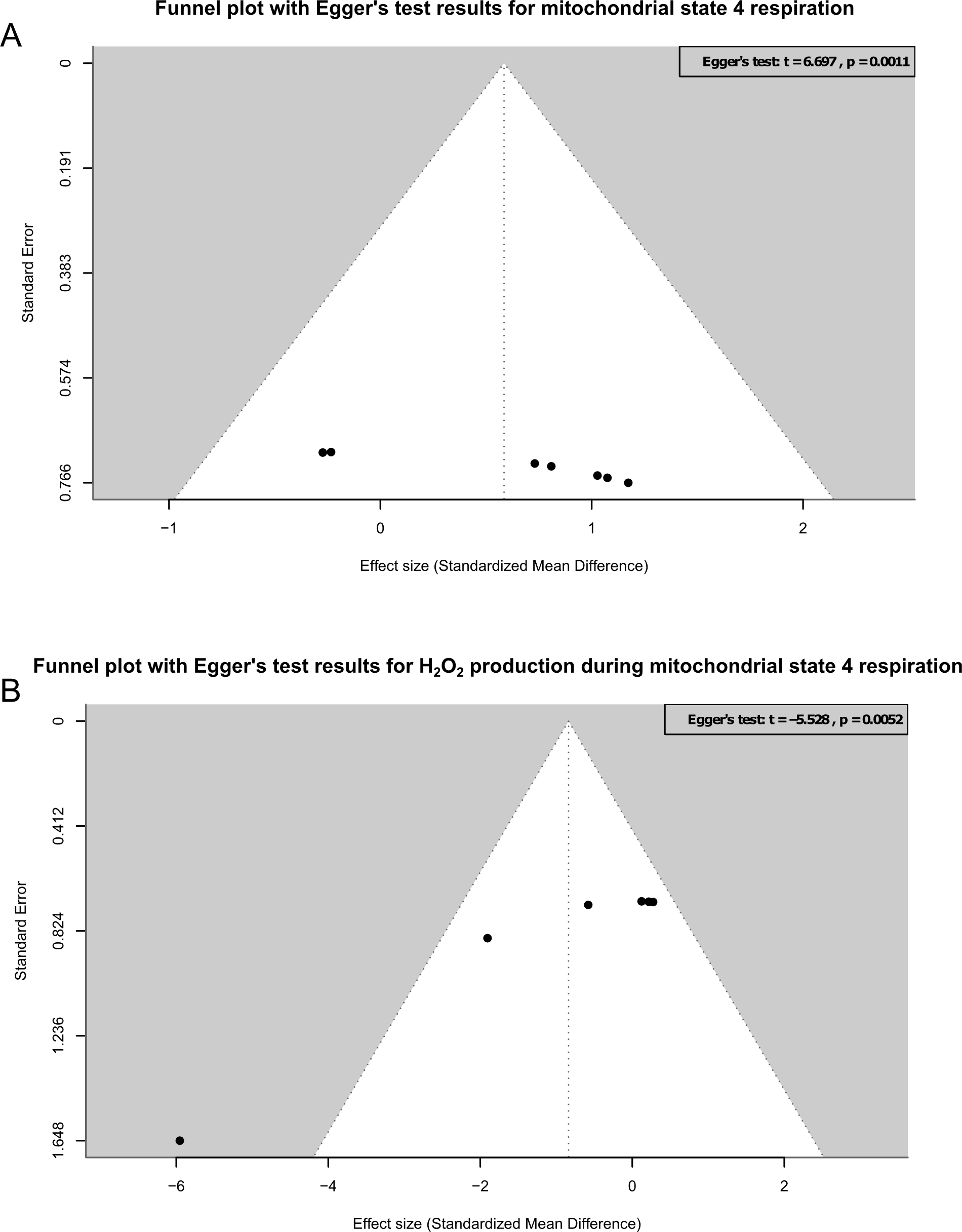


**Suppl. Fig. 7** Funnel plots for studies included in the analysis of mitochondrial respiration and H_2_O_2_ production during mitochondrial state 4 respiration. Funnel plots assessing the risk of publication bias in the studies that analyzed (A) mitochondrial respiration and (B) H_2_O_2_ production during mitochondrial state 4 respiration are presented. Each plot visualizes the distribution of studies effects and includes results from Egger's test to evaluate asymmetry, indicating potential bias (p < 0.05)


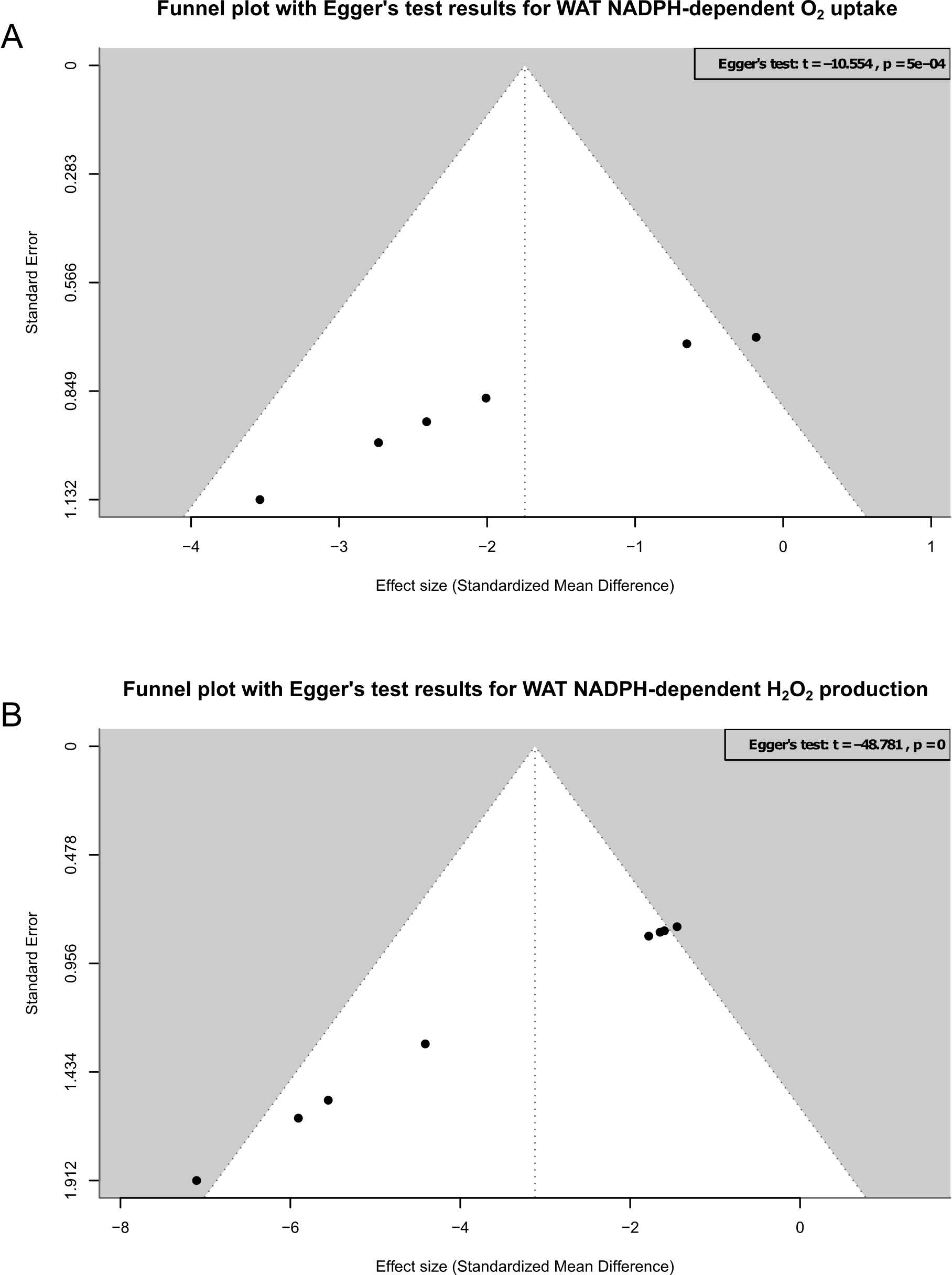


**Suppl. Fig. 8** Funnel plots for studies included in the analysis of NADPH-dependent O2 uptake and H_2_O_2_ production in white adipose tissue. Funnel plots assessing the risk of publication bias in the studies that analyzed (A) NADPH-dependent O_2_ uptake and (B) H_2_O_2_ production in white adipose tissue are presented. Each plot visualizes the distribution of studies effects and includes results from Egger's test to evaluate asymmetry, indicating potential bias (p < 0.05)


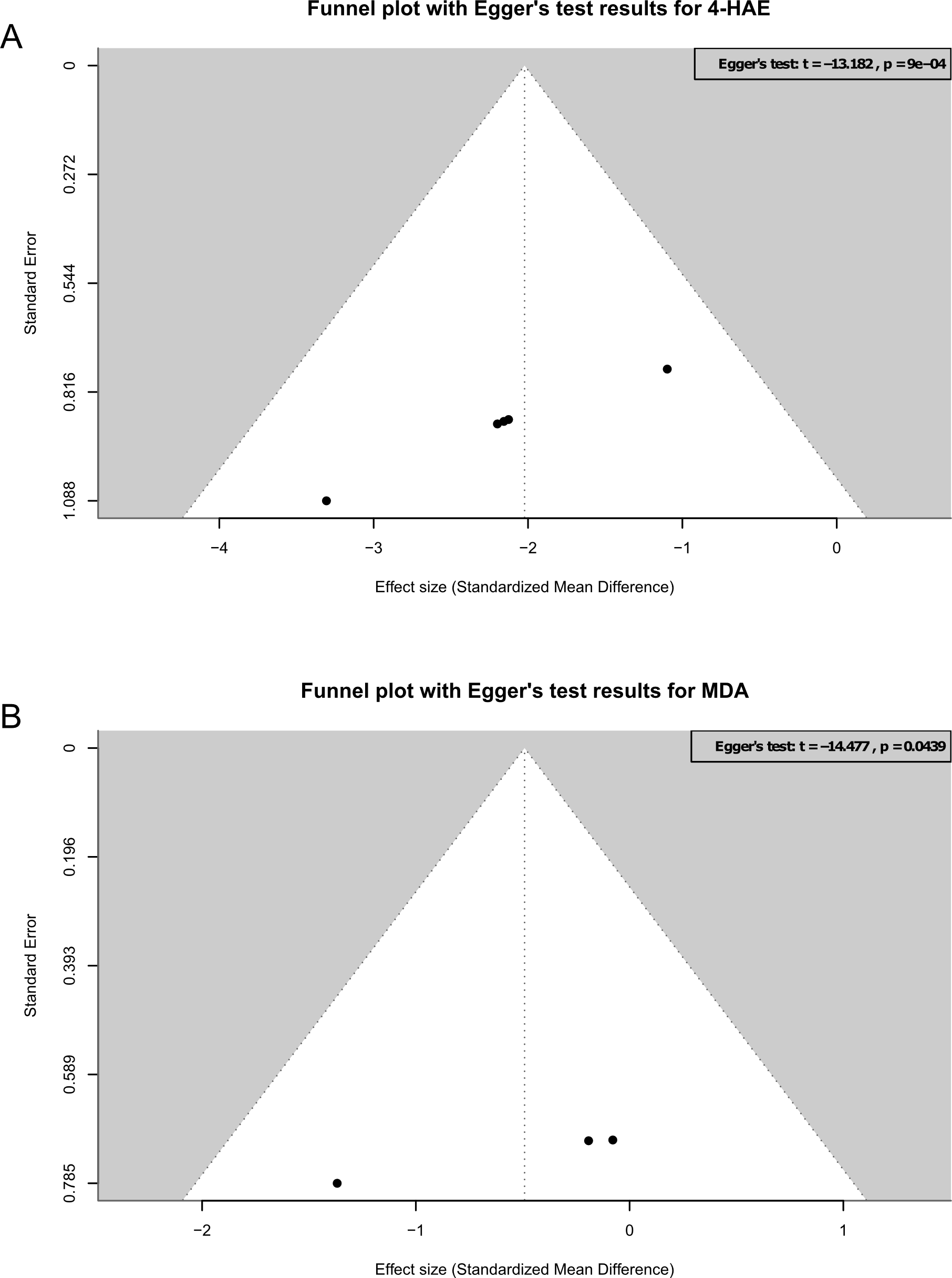


**Suppl. Fig. 9** Funnel plots for studies included in lipid peroxidation analysis. Funnel plots assessing the risk of publication bias in the studies that analyzed (A) 4-hydroxialkenals (4-HAE) levels and (B) malondialdehyde (MDA) levels are presented. Each plot visualizes the distribution of studies effects and includes results from Egger's test to evaluate asymmetry, indicating potential bias (p < 0.05)
