# Supplementary material for: Effects of tetrahydroindenoindole supplementation on metabolism: A systematic review with meta-analysis of rodent-based studies
Source: GeroScience. 2025 May 5;48(1):727–53. doi: 10.1007/s11357-025-01680-z (PMC12972266; doi:10.1007/s11357-025-01680-z)
Supplement: Supplementary file 2 — Supplementary file2 (DOCX 69 KB) [file 11357_2025_1680_MOESM2_ESM.docx]

**Suppl. Table 1** SYRCLE’s risk of bias tool for animal studies. Criteria: (1) Was the allocation sequence adequately generated and applied? (2) Were the groups similar at baseline or were they adjusted for confounders in the analysis? (3) Was the allocation to the different groups adequately concealed during? (4) Were the animals randomly housed during the experiments? (5) Were the caregivers and/or investigators blinded from knowledge which intervention each animal received during the experiments? (6) Were animals selected at random for outcome assessment? (7) Was the outcome assessor blinded? (8) Were incomplete outcome data adequately addressed? (9) Are reports of the study free of selective outcome reporting? (10) Was the study apparently free of other problems that could results in high risk of bias? Ref: references

| **Ref.** | **Study** | **(1)** | **(2)** | **(3)** | **(4)** | **(5)** | **(6)** | **(7)** | **(8)** | **(9)** | **(10)** | **Score** |
| --- | --- | --- | --- | --- | --- | --- | --- | --- | --- | --- | --- | --- |
| [24] | Shertzer and Sainsbury, 1991 | 0 | 0.75 | 0 | 0.75 | 0 | 0 | 0 | 1 | 1 | 0.70 | 4.20 |
| [7] | Shertzer *et al*., 2009 | 0 | 0.75 | 1 | 0.75 | 0 | 0 | 0 | 1 | 1 | 0.70 | 5.20 |
| [8] | Shertzer, 2010 | 0 | 0.75 | 0 | 0.75 | 0 | 0 | 0 | 1 | 1 | 0.70 | 4.20 |
| [9] | Shertzer *et al*., 2010 | 0 | 0.75 | 1 | 0.75 | 0 | 0 | 0 | 1 | 1 | 0.70 | 5.20 |
| [2] | Martin-Montalvo *et al*., 2016 | 1 | 0.75 | 1 | 0.75 | 0 | 0 | 0 | 1 | 1 | 0.80 | 6.30 |
| [3] | Watanabe *et al*., 2023 | 1 | 0.75 | 1 | 0.75 | 1 | 1 | 1 | 1 | 1 | 0.80 | 9.30 |
| [4] | Watanabe *et al*., 2024 | 0 | 0.75 | 0 | 0.75 | 0 | 0 | 0 | 1 | 1 | 0.80 | 4,30 |

**Suppl. Table 2** Adaptation of the CAMARADES checklist. Criteria: (1) peer reviewed publication; (2) control of temperature; (3) random allocation to treatment or control; (4) treatment dosage; (5) blinded assessment of outcome; (6) method of euthanasia; (7) animal model (strain, sex, age, experimental condition); (8) sample size calculation; (9) compliance with animal welfare regulations; and (10) statement of potential conflict of interests. Ref: references

| **Ref.** | **Study** | **(1)** | **(2)** | **(3)** | **(4)** | **(5)** | **(6)** | **(7)** | **(8)** | **(9)** | **(10)** | **Score** |
| --- | --- | --- | --- | --- | --- | --- | --- | --- | --- | --- | --- | --- |
| [24] | Shertzer and Sainsbury, 1991 | 1 | 1 | 0 | 1 | 0 | 1 | 1 | 0 | 0 | 0 | 5 |
| [7] | Shertzer *et al*., 2009 | 1 | 0 | 0 | 1 | 0 | 1 | 1 | 0 | 1 | 0 | 5 |
| [8] | Shertzer, 2010 | 1 | 0 | 0 | 1 | 0 | 1 | 1 | 0 | 1 | 1 | 6 |
| [9] | Shertzer *et al*., 2010 | 1 | 0 | 0 | 1 | 0 | 1 | 0 | 0 | 1 | 0 | 4 |
| [2] | Martin-Montalvo *et al*., 2016 | 1 | 1 | 1 | 1 | 0 | 1 | 1 | 0 | 1 | 1 | 8 |
| [3] | Watanabe *et al*., 2023 | 1 | 1 | 1 | 1 | 1 | 1 | 0 | 1 | 1 | 1 | 9 |
| [4] | Watanabe *et al*., 2024 | 1 | 1 | 0 | 1 | 0 | 1 | 0 | 0 | 1 | 1 | 6 |

**Suppl. Table 3** Sensitivity analysis results for body weight outcomes. The overall effect (see "Overall Effect" column) is presented before (see "No Exclusion" row) and after the exclusion of individual studies (see "Excluded Study" column and corresponding rows for the excluded studies). "Condition" column identifies the dietary patter associated with each study. SMD: standardized mean difference. 95% CI: 95% confidence interval.

|  |  | Overall effect | |
| --- | --- | --- | --- |
| Excluded study | Condition | SMD [95% CI] | p-value |
| No exclusion | - | -0.31 [-1.32, 0.70] | 0.5491 |
| Shertzer *et al*., 2010 | High-fat diet | 0.15 [-0.60, 0.90] | 0.6915 |
| Shertzer *et a*l., 2009 | Normal diet | -0.41 [-1.84, 1.02] | 0.5744 |
| Shertzer *et a*l., 2010 | Normal diet | -0.35 [-1.77, 1.08] | 0.6322 |
| Watanabe *et al*., 2023 | Normal diet | -0.70 [-1.72, 0.32] | 0.1774 |

**Suppl. Table 4** Sensitivity analysis results for body weight gain outcomes. The table presents the results of the sensitivity analysis for body weight gain outcomes, showing both subgroup effects (see "Subgroup Effect" column) and overall effects (see "Overall Effect" column). The effect is presented before (see "No Exclusion" row) and after the exclusion of individual studies (see "Excluded Study" column and corresponding rows for the excluded studies). "Subgroup" column identifies the variable used for the subgroup analysis, and each entry corresponds to the subgroup associated with each result. "NA" indicates values that are not available after excluding individual studies, as subgroups were only considered when at least two studies were included in a subgroup. SMD: standardized mean difference. 95% CI: 95% confidence interval.

|  |  | Subgroup effect | | Overall effect | |
| --- | --- | --- | --- | --- | --- |
| Excluded study | Subgroup | SMD [95% CI] | p-value | SMD [95% CI] | p-value |
| No exclusion | Normal diet | -0.45 [-1.01, 0.11] | 0.1122 | -0.67 [-1.16, -0.17] | 0.0089 |
|  | High-fat diet | -1.57 [-2.70, -0.43] | 0.0069 |  |  |
| Watanabe *et al*., 2023 | Normal diet | -0.41 [-1.04, 0.22] | 0.2059 | -0.71 [-1.31, -0.11] | 0.0205 |
| Shertzer *et al*., 2010 | Normal diet | -0.50 [-1.11, 0.10] | 0.1043 | -0.75 [-1.30, -0.20] | 0.0077 |
| Shertzer, 2010 | Normal diet | -0.31 [-0.91, 0.28] | 0.3048 | -0.58 [-1.11, -0.05] | 0.0305 |
| Shertzer *et a*l., 2009 | Normal diet | -0.50 [-1.11, 0.10] | 0.1034 | -0.75 [-1.30, 1.20] | 0.0076 |
| Shertzer *et al*., 2009 | Normal diet | -0.54 [-1.22, 0.13] | 0.1132 | -0.81 [-1.39, -0.23] | 0.0061 |
| Shertzer *et al*., 2010 | High-fat diet | NA | NA | -0.53 [-1.05, -0.01] | 0.0458 |
| Shertzer *et al*., 2009 | High-fat diet | NA | NA | -0.62 [-1.18, -0.07] | 0.0280 |

**Suppl. Table 5** Sensitivity analysis results for body fat outcomes. The table presents the results of the sensitivity analysis for body fat outcomes, showing both subgroup effects (see "Subgroup Effect" column) and overall effects (see "Overall Effect" column). The effect is presented before (see "No Exclusion" row) and after the exclusion of individual studies (see "Excluded Study" column and corresponding rows for the excluded studies). "Subgroup" column identifies the variable used for the subgroup analysis, and each entry corresponds to the subgroup associated with each result. "NA" indicates values that are not available after excluding individual studies, as subgroups were only considered when at least two studies were included in a subgroup. SMD: standardized mean difference. 95% CI: 95% confidence interval.

|  |  | Subgroup effect | | Overall effect | |
| --- | --- | --- | --- | --- | --- |
| Excluded study | Subgroup | SMD [95% CI] | p-value | SMD [95% CI] | p-value |
| No exclusion | Normal diet | 0.24 [-0.57, 1.04] | 0.5628 | -0.10 [-0.74, 0.53] | 0.7458 |
|  | High-fat diet | -0.66 [-1.68, 0.53] | 0.2082 |  |  |
| Shertzer *et al*., 2010 | Normal diet | 0.27 [-0.72, 1.26] | 0.5914 | -0.18 [-0.89, 0.53] | 0.6254 |
| Shertzer, 2010 | Normal diet | 0.16 [-0.82, 1.14] | 0.7529 | -0.23 [-0.94, 0.47] | 0.5185 |
| Shertzer *et al*., 2009 | Normal diet | 0.28 [-0.70, 1.27] | 0.5710 | -0.17 [-0.88, 0.54] | 0.6405 |
| Shertzer *et al*., 2010 | High-fat diet | NA | NA | 0.13 [-0.57, 0.82] | 0.7192 |
| Shertzer *et al*., 2009 | High-fat diet | NA | NA | -0.08 [-0.79, 0.63] | 0.8261 |

**Suppl. Table 6** Sensitivity analysis results for fasting blood glucose outcomes. The table presents the results of the sensitivity analysis for fasting blood glucose outcomes, showing both subgroup effects (see "Subgroup Effect" column) and overall effects (see "Overall Effect" column). The effect is presented before (see "No Exclusion" row) and after the exclusion of individual studies (see "Excluded Study" column and corresponding rows for the excluded studies). "Subgroup" column identifies the variable used for the subgroup analysis, and each entry corresponds to the subgroup associated with each result. "NA" indicates values that are not available after excluding individual studies, as subgroups were only considered when at least two studies were included in a subgroup. SMD: standardized mean difference. 95% CI: 95% confidence interval.

|  |  | Subgroup effect | | Overall effect | |
| --- | --- | --- | --- | --- | --- |
| Excluded study | Subgroup | SMD [95% CI] | p-value | SMD [95% CI] | p-value |
| No exclusion | Normal diet | -0.35 [-1.09, 0.40] | 0.3629 | -0.49 [-1.09, 0.12] | 0.1131 |
|  | High-fat diet | -0.74 [-1.76, 0.27] | 0.1507 |  |  |
| Watanabe *et al*., 2024 | Normal diet | -0.36 [-1.35, 0.63] | 0.4764 | -0.55 [-1.25, 0.16] | 0.1304 |
| Shertzer *et al*., 2010 | Normal diet | -0.32 [-1.20, 0.56] | 0.4743 | -0.50 [-1.17, 0.16] | 0.1381 |
| Shertzer *et al*., 2009 | Normal diet | -0.36 [-1.24, 0.52] | 0.4232 | -0.53 [-1.19, 0.14] | 0.1217 |
| Shertzer *et al*., 2010 | High-fat diet | NA | NA | -0.40 [-1.06, 0.26] | 0.2390 |
| Shertzer *et al*., 2009 | High-fat diet | NA | NA | -0.47 [-1.13, 0.20] | 0.1690 |

**Suppl. Table 7** Sensitivity analysis results for glucose levels during glucose tolerance test outcomes. The table presents the results of the glucose levels during glucose tolerance test outcomes, showing both subgroup effects (see "Subgroup Effect" column) and overall effects (see "Overall Effect" column). The effect is presented before (see "No Exclusion" row) and after the exclusion of individual studies (see "Excluded Study" column and corresponding rows for the excluded studies). "Subgroup" column identifies the variable used for the subgroup analysis, and each entry corresponds to the subgroup associated with each result. "NA" indicates values that are not available after excluding individual studies, as subgroups were only considered when at least two studies were included in a subgroup. SMD: standardized mean difference. 95% CI: 95% confidence interval.

|  |  | Subgroup effect | | Overall effect | |
| --- | --- | --- | --- | --- | --- |
| Excluded study | Subgroup | SMD [95% CI] | p-value | SMD [95% CI] | p-value |
| No exclusion | Normal diet | -0.25 [-0.88, 0.38] | 0.4323 | -0.87 [-1.86, 0.12] | 0.0851 |
|  | High-fat diet | -2.77 [-4.16, -1.38] | <0.0001 |  |  |
| Watanabe *et al*., 2024 | Normal diet | -0.21 [-0.97, 0.57] | 0.5980 | -1.02 [-2.24, 0.19] | 0.0995 |
| Shertzer *et al*., 2010 | Normal diet | -0.20 [-0.98, 0.57] | 0.6086 | -1.02 [-2.24, 0.20] | 0.1014 |
| Shertzer *et al*., 2009 | Normal diet | -0.49 [-1.19, 0.22] | 0.1737 | -1.17 [-2.18, -0.17] | 0.0222 |
| Shertzer *et al*., 2009 | Normal diet | -0.00 [-0.81, 0.81] | 0.9990 | -0.99 [-2.31, 0.33] | 0.1424 |
| Shertzer *et al*., 2010 | High-fat diet | NA | NA | -0.62 [-1.63, 0.40] | 0.2329 |
| Shertzer *et al*., 2009 | High-fat diet | NA | NA | -0.50 [-1.29, 0.30] | 0.2197 |

**Suppl. Table 8** Sensitivity analysis results for fasting blood insulin outcomes. The table presents the results of the fasting blood insulin outcomes, showing both subgroup effects (see "Subgroup Effect" column) and overall effects (see "Overall Effect" column). The effect is presented before (see "No Exclusion" row) and after the exclusion of individual studies (see "Excluded Study" column and corresponding rows for the excluded studies). "Subgroup" column identifies the variable used for the subgroup analysis, and each entry corresponds to the subgroup associated with each result. "NA" indicates values that are not available after excluding individual studies, as subgroups were only considered when at least two studies were included in a subgroup. "NA" indicates values that are not available after excluding individual studies, as subgroups were only considered when at least two studies were included in a subgroup. SMD: standardized mean difference. 95% CI: 95% confidence interval.

|  |  | Subgroup effect | | Overall effect | |
| --- | --- | --- | --- | --- | --- |
| Excluded study | Subgroup | SMD [95% CI] | p-value | SMD [95% CI] | p-value |
| No exclusion | Normal diet | 0.54 [-0.22, 1.30] | 0.1648 | -0.01 [-0.77, 0.76] | 0.9886 |
|  | High-fat diet | -0.90 [-1.94, 0.14] | 0.0898 |  |  |
| Watanabe *et al*., 2024 | Normal diet | 0.28 [-0.71, 1.26] | 0.5792 | -0.28 [-1.04, 0.47] | 0.4620 |
| Shertzer *et al*., 2010 | Normal diet | 0.65 [-0.25, 1.56] | 0.1582 | -0.10 [-1.07, 0.88] | 0.8472 |
| Shertzer *et al*., 2009 | Normal diet | 0.64 [-0.26, 1.54] | 0.1639 | -0.10 [-1.08, 0.88] | 0.8394 |
| Shertzer *et al*., 2010 | High-fat diet | NA | NA | 0.31 [-0.36, 0.97] | 0.3671 |
| Shertzer *et al*., 2009 | High-fat diet | NA | NA | 0.10 [-0.84, 1.03] | 0.8406 |

**Suppl. Table 9** Sensitivity analysis results for insulin levels during glucose tolerance test outcomes. The table presents the results of the insulin levels during glucose tolerance test outcomes, showing both subgroup effects (see "Subgroup Effect" column) and overall effects (see "Overall Effect" column). The effect is presented before (see "No Exclusion" row) and after the exclusion of individual studies (see "Excluded Study" column and corresponding rows for the excluded studies). "Subgroup" column identifies the variable used for the subgroup analysis, and each entry corresponds to the subgroup associated with each result. "NA" indicates values that are not available after excluding individual studies, as subgroups were only considered when at least two studies were included in a subgroup. "NA" indicates values that are not available after excluding individual studies, as subgroups were only considered when at least two studies were included in a subgroup. SMD: standardized mean difference. 95% CI: 95% confidence interval.

|  |  | Subgroup effect | | Overall effect | |
| --- | --- | --- | --- | --- | --- |
| Excluded study | Subgroup | SMD [95% CI] | p-value | SMD [95% CI] | p-value |
| No exclusion | Normal diet | 0.32 [-0.49, 1.13] | 0.4405 | 0.36 [-0.27, 0.99] | 0.2584 |
|  | High-fat diet | 0.43 [-0.57, 1.42] | 0.3993 |  |  |
| Watanabe *et al*., 2024 | Normal diet | 0.22 [-0.76, 1.21] | 0.6574 | 0.32 [-0.38, 1.03] | 0.3635 |
| Shertzer *et al*., 2010 | Normal diet | 0.23 [-0.76, 1.22] | 0.6512 | 0.33 [-0.37, 1.03] | 0.3602 |
| Shertzer *et al*., 2009 | Normal diet | 0.51 [-0.49, 1.50] | 0.3190 | 0.47 [-0.24, 1.17] | 0.1934 |
| Shertzer *et al*., 2010 | High-fat diet | NA | NA | 0.39 [-0.31, 1.10] | 0.2722 |
| Shertzer *et al*., 2009 | High-fat diet | NA | NA | 0.30 [-0.40, 1.00] | 0.4041 |

**Suppl. Table 10** Sensitivity analysis results for O_2_ consumption outcomes. The overall effect (see "Overall Effect" column) is presented before (see "No Exclusion" row) and after the exclusion of individual studies (see "Excluded Study" column and corresponding rows for the excluded studies). "Condition" column identifies the dietary patter associated with each study. SMD: standardized mean difference. 95% CI: 95% confidence interval.

|  |  | Overall effect | |
| --- | --- | --- | --- |
| Excluded study | Condition | SMD [95% CI] | p-value |
| No exclusion | - | 2.79 [1.65, 3.93] | <0.0001 |
| Shertzer *et al*., 2010 | High-fat diet | 2.49 [1.18, 3.81] | 0.0002 |
| Shertzer *et al*., 2009 | Normal diet | 3.36 [1.84, 4.89] | <0.0001 |
| Shertzer *et al*., 2010 | Normal diet | 2.68 [1.16, 4.21] | 0.0006 |

**Suppl. Table 11** Sensitivity analysis results for CO_2_ production outcomes. The overall effect (see "Overall Effect" column) is presented before (see "No Exclusion" row) and after the exclusion of individual studies (see "Excluded Study" column and corresponding rows for the excluded studies). "Condition" column identifies the dietary patter associated with each study. SMD: standardized mean difference. 95% CI: 95% confidence interval.

|  |  | Overall effect | |
| --- | --- | --- | --- |
| Excluded study | Condition | SMD [95% CI] | p-value |
| No exclusion | - | 1.54 [0.62, 2.46] | 0.0010 |
| Shertzer *et al*., 2010 | High-fat diet | 1.30 [0.22, 2.38] | 0.0182 |
| Shertzer *et al*., 2009 | Normal diet | 1.75 [0.59, 2.91] | 0.0031 |
| Shertzer *et al*., 2010 | Normal diet | 1.60 [0.46, 2.74] | 0.0058 |

**Suppl. Table 12** Sensitivity analysis results for respiratory quotient outcomes. The overall effect (see "Overall Effect" column) is presented before (see "No Exclusion" row) and after the exclusion of individual studies (see "Excluded Study" column and corresponding rows for the excluded studies). "Condition" column identifies the dietary patter associated with each study. SMD: standardized mean difference. 95% CI: 95% confidence interval.

|  |  | Overall effect | |
| --- | --- | --- | --- |
| Excluded study | Condition | SMD [95% CI] | p-value |
| No exclusion | - | -2.02 [-4.08, 0.04] | 0.0549 |
| Shertzer *et al*., 2010 | High-fat diet | -3.07 [-6.72, 0.61] | 0.1021 |
| Shertzer *et al*., 2009 | Normal diet | -0.98 [-2.03, 0.06] | 0.0659 |
| Shertzer *et al*., 2010 | Normal diet | -2.71 [-7.11, 1.68] | 0.2267 |

**Suppl. Table 13** Sensitivity analysis results for mitochondrial state 3 respiration outcomes. The overall effect (see "Overall Effect" column) is presented before (see "No Exclusion" row) and after the exclusion of individual studies (see "Excluded Study" column and corresponding rows for the excluded studies). "Condition" column identifies the dietary patter associated with each study. SMD: standardized mean difference. 95% CI: 95% confidence interval.

|  |  | Overall effect | |
| --- | --- | --- | --- |
| Excluded study | Condition | SMD [95% CI] | p-value |
| No exclusion | - | 0.07 [-0.73, 0.87] | 0.8601 |
| Shertzer *et al*., 2009 | High-fat diet | -0.05 [-1.03, 0.93] | 0.9175 |
| Shertzer *et al*., 2009 | Normal diet | 0.21 [-0.78, 1.19] | 0.6803 |
| Shertzer, 2010 | Normal diet | 0.06 [-0.92, 1.05] | 0.9016 |

**Suppl. Table 14** Sensitivity analysis results for mitochondrial state 4 respiration outcomes. The overall effect (see "Overall Effect" column) is presented before (see "No Exclusion" row) and after the exclusion of individual studies (see "Excluded Study" column and corresponding rows for the excluded studies). "Condition" column identifies the dietary patter associated with each study. SMD: standardized mean difference. 95% CI: 95% confidence interval.

|  |  | Overall effect | |
| --- | --- | --- | --- |
| Excluded study | Condition | SMD [95% CI] | p-value |
| No exclusion | - | 0.85 [0.02, 1.69] | 0.0458 |
| Shertzer *et al*., 2009 | High-fat diet | 0.92 [-0.12, 1.95] | 0.0817 |
| Shertzer *et al*., 2009 | Normal diet | 0.87 [-0.15, 1.90] | 0.0952 |
| Shertzer, 2010 | Normal diet | 0.77 [-0.25, 1.79] | 0.1376 |

**Suppl. Table 15** Sensitivity analysis results for respiratory control ratio respiration outcomes. The overall effect (see "Overall Effect" column) is presented before (see "No Exclusion" row) and after the exclusion of individual studies (see "Excluded Study" column and corresponding rows for the excluded studies). "Condition" column identifies the dietary patter associated with each study. SMD: standardized mean difference. 95% CI: 95% confidence interval.

|  |  | Overall effect | |
| --- | --- | --- | --- |
| Excluded study | Condition | SMD [95% CI] | p-value |
| No exclusion | - | -1.13 [-2.00, -0.26] | 0.0106 |
| Shertzer *et al*., 2009 | High-fat diet | -1.40 [-2.50, -0.31] | 0.0122 |
| Shertzer *et al*., 2009 | Normal diet | -1.10 [-2.17, -0.04] | 0.0421 |
| Shertzer, 2010 | Normal diet | -0.92 [-1.96, 0.11] | 0.0803 |

**Suppl. Table 16** Sensitivity analysis results for ATP levels outcomes. The overall effect (see "Overall Effect" column) is presented before (see "No Exclusion" row) and after the exclusion of individual studies (see "Excluded Study" column and corresponding rows for the excluded studies). "Condition" column identifies the dietary patter associated with each study. SMD: standardized mean difference. 95% CI: 95% confidence interval.

|  |  | Overall effect | |
| --- | --- | --- | --- |
| Excluded study | Condition | SMD [95% CI] | p-value |
| No exclusion | - | 0.08 [-0.72, 0.89] | 0.8384 |
| Shertzer *et al*., 2009 | High-fat diet | 0.01 [-0.98, 0.99] | 0.9882 |
| Shertzer *et al*., 2010 | Normal diet | 0.02 [-0.96, 1.00] | 0.9653 |
| Shertzer *et al*., 2009 | Normal diet | 0.22 [-0.76, 1.20] | 0.6590 |

**Suppl. Table 17** Sensitivity analysis results for mitochondrial state 4 respiration outcomes with normal diet/high-fat diet subgroups. The table presents the results of the mitochondrial state 4 respiration outcomes with normal diet/high-fat diet subgroups, showing both subgroup effects (see "Subgroup Effect" column) and overall effects (see "Overall Effect" column). The effect is presented before (see "No Exclusion" row) and after the exclusion of individual studies (see "Excluded Study" column and corresponding rows for the excluded studies). "Subgroup" column identifies the variable used for the subgroup analysis, and each entry corresponds to the subgroup associated with each result. “Tissue” column identifies the tissue associated with each study. SMD: standardized mean difference. 95% CI: 95% confidence interval.

|  |  |  | Subgroup effect | | Overall effect | |
| --- | --- | --- | --- | --- | --- | --- |
| Excluded study | Tissue | Subgroup | SMD [95% CI] | p-value | SMD [95% CI] | p-value |
| No exclusion | - | Normal diet | 0.63 [-0.09, 1.36] | 0.0863 | 0.59 [0.04, 1.13] | 0.0355 |
|  | - | High-fat diet | 0.52 [-0.31, 1.35] | 0.2177 |  |  |
| Shertzer *et al*., 2010 | WAT | Normal diet | 0.50 [-0.33, 1.32] | 0.2416 | 0.51 [-0.08, 1.10] | 0.0892 |
| Shertzer *et al*., 2009 | WAT | Normal diet | 0.97 [0.12, 1.81] | 0.0251 | 0.74 [0.15, 1.33] | 0.0143 |
| Shertzer, 2010 | Liver | Normal diet | 0.51 [-0.32, 1.34] | 0.2303 | 0.52 [-0.07, 1.10] | 0.0855 |
| Shertzer *et al*., 2009 | Liver | Normal diet | 0.58 [-0.32, 1.47] | 0.2049 | 0.55 [-0.04, 1.14] | 0.0684 |
| Shertzer *et al*., 2010 | WAT | High-fat diet | 0.23 [-0.76, 1.23] | 0.6446 | 0.50 [-0.09, 1.08] | 0.0971 |
| Shertzer *et al*., 2009 | WAT | High-fat diet | 0.94 [-0.09, 1.98] | 0.0748 | 0.73 [0.14, 1.33] | 0.0153 |
| Shertzer *et al*., 2009 | Liver | High-fat diet | 0.44 [-0.94, 1.82] | 0.5305 | 0.56 [-0.03, 1.15] | 0.0626 |

**Suppl. Table 18** Sensitivity analysis results for mitochondrial state 4 respiration outcomes with white adipose tissue/liver subgroups. The table presents the results of the mitochondrial state 4 respiration outcomes with normal diet/high-fat diet subgroups, showing both subgroup effects (see "Subgroup Effect" column) and overall effects (see "Overall Effect" column). The effect is presented before (see "No Exclusion" row) and after the exclusion of individual studies (see "Excluded Study" column and corresponding rows for the excluded studies). "Subgroup" column identifies the variable used for the subgroup analysis, and each entry corresponds to the subgroup associated with each result. "Condition" column identifies the dietary patter associated with each study. SMD: standardized mean difference. 95% CI: 95% confidence interval.

|  |  |  | Subgroup effect | | Overall effect | |
| --- | --- | --- | --- | --- | --- | --- |
| Excluded study | Condition | Subgroup | SMD [95% CI] | p-value | SMD [95% CI] | p-value |
| No exclusion | - | WAT | 0.39 [-0.38, 1.17] | 0.3199 | 0.59 [0.04, 1.13] | 0.0355 |
|  | - | Liver | 0.85 [0.02, 1.69] | 0.0458 |  |  |
| Shertzer *et al*., 2010 | High-fat diet | WAT | 0.15 [-0.68, 0.98] | 0.7164 | 0.50 [-0.09, 1.08] | 0.0971 |
| Shertzer *et al*., 2010 | Normal diet | WAT | 0.18 [-0.71, 1.07] | 0.6879 | 0.51 [-0.08, 1.10] | 0.0892 |
| Shertzer *et al*., 2009 | High-fat diet | WAT | 0.62 [-0.32, 1.57] | 0.1957 | 0.73 [0.14, 1.33] | 0.0153 |
| Shertzer *et al*., 2009 | Normal diet | WAT | 0.64 [-0.28, 1.56] | 0.1760 | 0.74 [0.15, 1.33] | 0.0143 |
| Shertzer, 2010 | Normal diet | Liver | 0.77 [-0.25, 1.79] | 0.1376 | 0.52 [-0.07, 1.10] | 0.0855 |
| Shertzer *et al*., 2009 | High-fat diet | Liver | 0.92 [-0.12, 1.95] | 0.0817 | 0.56 [-0.03, 1.15] | 0.0626 |
| Shertzer *et al*., 2009 | Normal diet | Liver | 0.87 [-0.15, 1.90] | 0.0952 | 0.55 [-0.04, 1.14] | 0.0684 |

**Suppl. Table 19** Sensitivity analysis results for H_2_O_2_ production during mitochondrial state 4 respiration outcomes with normal diet/high-fat diet subgroups. The table presents the results of the H_2_O_2_ production during mitochondrial state 4 respiration outcomes with normal diet/high-fat diet subgroups, showing both subgroup effects (see "Subgroup Effect" column) and overall effects (see "Overall Effect" column). The effect is presented before (see "No Exclusion" row) and after the exclusion of individual studies (see "Excluded Study" column and corresponding rows for the excluded studies). "Subgroup" column identifies the variable used for the subgroup analysis, and each entry corresponds to the subgroup associated with each result. “Tissue” column identifies the tissue associated with each study. "NA" indicates values that are not available after excluding individual studies, as subgroups were only considered when at least two studies were included in a subgroup. SMD: standardized mean difference. 95% CI: 95% confidence interval.

|  |  |  | Subgroup effect | | Overall effect | |
| --- | --- | --- | --- | --- | --- | --- |
| Excluded study | Tissue | Subgroup | SMD [95% CI] | p-value | SMD [95% CI] | p-value |
| No exclusion | - | Normal diet | -1.73 [-4.04, 0.58] | 0.1412 | -0.84 [-2.03, 0.36] | 0.1695 |
|  | - | High-fat diet | 0.20 [-0.78, 1.18] | 0.6922 |  |  |
| Shertzer *et al*., 2009 | Liver | Normal diet | -0.68 [-1.84, 0.48] | 0.2493 | -0.29 [-1.03, 0.45] | 0.4387 |
| Shertzer, 2010 | Liver | Normal diet | -1.83 [-5.35, 1.68] | 0.3058 | -0.62 [-1.93, 0.69] | 0.3512 |
| Shertzer *et al*., 2010 | WAT | Normal diet | -2.29 [-5.63, 1.06] | 0.1803 | -0.98 [-2.49, 0.53] | 0.2022 |
| Shertzer *et al*., 2009 | WAT | Normal diet | -2.50 [-5.38, 0.37] | 0.0880 | -1.14 [-2.60, 0.32] | 0.1255 |
| Shertzer *et al*., 2010 | WAT | High-fat diet | NA | NA | -1.12 [-2.60, 0.35] | 0.1344 |
| Shertzer *et al*., 2009 | WAT | High-fat diet | NA | NA | -1.15 [-2.59, 0.30] | 0.1201 |

**Suppl. Table 20** Sensitivity analysis results for H_2_O_2_ production mitochondrial state 4 respiration outcomes with white adipose tissue/liver subgroups. The table presents the results of the H_2_O_2_ production mitochondrial state 4 respiration outcomes with white adipose tissue/liver subgroups, showing both subgroup effects (see "Subgroup Effect" column) and overall effects (see "Overall Effect" column). The effect is presented before (see "No Exclusion" row) and after the exclusion of individual studies (see "Excluded Study" column and corresponding rows for the excluded studies). "Subgroup" column identifies the variable used for the subgroup analysis, and each entry corresponds to the subgroup associated with each result. "Condition" column identifies the dietary patter associated with each study. "NA" indicates values that are not available after excluding individual studies, as subgroups were only considered when at least two studies were included in a subgroup. SMD: standardized mean difference. 95% CI: 95% confidence interval.

|  |  |  | Subgroup effect | | Overall effect | |
| --- | --- | --- | --- | --- | --- | --- |
| Excluded study | Condition | Subgroup | SMD [95% CI] | p-value | SMD [95% CI] | p-value |
| No exclusion | - | WAT | 0.01 [-0.68, 0.71] | 0.9689 | -0.84 [-2.03, 0.36] | 0.1695 |
|  | - | Liver | -3.68 [-7.62, 0.26] | 0.0668 |  |  |
| Shertzer *et al*., 2010 | High-fat diet | WAT | -0.02 [-0.83, 0.78] | 0.9554 | -1.12 [-2.60, 0.35] | 0.1344 |
| Shertzer *et al*., 2010 | Normal diet | WAT | 0.20 [-0.60, 1.01] | 0.6171 | -0.98 [-2.49, 0.53] | 0.2022 |
| Shertzer *et al*., 2009 | High-fat diet | WAT | -0.07 [-0.88, 0.73] | 0.8581 | -1.15 [-2.59, 0.30] | 0.1201 |
| Shertzer *et a*l., 2009 | Normal diet | WAT | -0.05 [-0.86, 0.75] | 0.8945 | -1.14 [-2.60, 0.32] | 0.1255 |
| Shertzer *et al*., 2009 | Normal diet | Liver | NA | NA | -0.29 [-1.03, 0.45] | 0.4387 |
| Shertzer, 2010 | Normal diet | Liver | NA | NA | -0.62 [-1.93, 0.69] | 0.3512 |

**Suppl. Table 21** Sensitivity analysis results for NADPH-dependent O_2_ uptake outcomes with NADPH+DPI/NADPH subgroups. The table presents the results of the NADPH-dependent O_2_ uptake outcomes with NADPH+DPI/NADPH subgroups, showing both subgroup effects (see "Subgroup Effect" column) and overall effects (see "Overall Effect" column). The effect is presented before (see "No Exclusion" row) and after the exclusion of individual studies (see "Excluded Study" column and corresponding rows for the excluded studies). "Subgroup" column identifies the variable used for the subgroup analysis, and each entry corresponds to the subgroup associated with each result. "Condition" column identifies the dietary patter associated with each study. "NA" indicates values that are not available after excluding individual studies, as subgroups were only considered when at least two studies were included in a subgroup. SMD: standardized mean difference. 95% CI: 95% confidence interval.

|  |  |  | Subgroup effect | | Overall effect | |
| --- | --- | --- | --- | --- | --- | --- |
| Excluded study | Condition | Subgroup | SMD [95% CI] | p-value | SMD [95% CI] | p-value |
| No exclusion | - | NADPH+DPI | -0.41 [-1.40, 0.58] | 0.4174 | -1.74 [-2.78, -0.71] | 0.0009 |
|  | - | NADPH | -2.57 [-3.51, -1.62] | <0.0001 |  |  |
| Shertzer *et al*., 2010 | High-fat diet | NADPH+DPI | NA | NA | -2.02 [-3.21, -0.83] | 0.0009 |
| Shertzer *et al*., 2010 | Normal diet | NADPH+DPI | NA | NA | -2.08 [-3.06, -1.10] | <0.0001 |
| Shertzer *et al*., 2010 | High-fat diet | NADPH | -2.82 [-3.95, -1.68] | <0.0001 | -1.73 [-2.97, -0.48] | 0.0066 |
| Shertzer *et al*., 2010 | Normal diet | NADPH | -2.52 [-3.60, -1.43] | <0.0001 | -1.57 [-2.71, -0.44] | 0.0066 |
| Shertzer *et al*., 2009 | High-fat diet | NADPH | -2.63 [-3.73, -1.52] | <0.0001 | -1.64 [-2.83, -0.45] | 0.0069 |
| Shertzer *et al*., 2009 | Normal diet | NADPH | -2.35 [-3.40, -1.31] | <0.0001 | -1.46 [-2.46, -0.46] | 0.0042 |

**Suppl. Table 22** Sensitivity analysis results for NADPH-dependent H_2_O_2_ production outcomes with NADPH+DPI/NADPH subgroups. The table presents the results of the NADPH-dependent H_2_O_2_ production outcomes with NADPH+DPI/NADPH subgroups, showing both subgroup effects (see "Subgroup Effect" column) and overall effects (see "Overall Effect" column). The effect is presented before (see "No Exclusion" row) and after the exclusion of individual studies (see "Excluded Study" column and corresponding rows for the excluded studies). "Subgroup" column identifies the variable used for the subgroup analysis, and each entry corresponds to the subgroup associated with each result. "Condition" column identifies the dietary patter associated with each study. SMD: standardized mean difference. 95% CI: 95% confidence interval.

|  |  |  | Subgroup effect | | Overall effect | |
| --- | --- | --- | --- | --- | --- | --- |
| Excluded study | Condition | Subgroup | SMD [95% CI] | p-value | SMD [95% CI] | p-value |
| No exclusion | - | NADPH+DPI | -1.61 [-2.41, -0.82] | <0.0001 | -3.12 [-4.42, -1.82] | <0.0001 |
|  | - | NADPH | -5.49 [-7.02, -3.96] | <0.0001 |  |  |
| Shertzer *et al*., 2010 | High-fat diet | NADPH+DPI | -1.56 [-2.48, -0.65] | 0.0008 | -3.45 [-4.99, -1.90] | <0.0001 |
| Shertzer *et al*., 2010 | Normal diet | NADPH+DPI | -1.62 [-2.54, -0.70] | 0.0006 | -3.47 [-5.01, -1.94] | <0.0001 |
| Shertzer *et al*., 2009 | High-fat diet | NADPH+DPI | -1.67 [-2.60, -0.74] | 0.0004 | -3.49 [-5.01, -1.98] | <0.0001 |
| Shertzer *et al*., 2009 | Normal diet | NADPH+DPI | -1.60 [-2.52, -0.68] | 0.0006 | -3.47 [-5.00, -1.93] | <0.0001 |
| Shertzer *et al*., 2010 | High-fat diet | NADPH | -5.16 [-6.84, -3.49] | <0.0001 | -2.70 [-3.87, -1.51] | <0.0001 |
| Shertzer *et al*., 2010 | Normal diet | NADPH | -5.46 [-7.23, -3.70] | <0.0001 | -2.81 [-4.10, -1.52] | <0.0001 |
| Shertzer *et al*., 2009 | High-fat diet | NADPH | -5.36 [-7.10, -3.62] | <0.0001 | -2.78 [-4.04, -1.51] | <0.0001 |
| Shertzer *et al*., 2009 | Normal diet | NADPH | -6.08 [-7.98, -4.17] | <0.0001 | -2.95 [-4.34, -1.57] | <0.0001 |

**Suppl. Table 23** Sensitivity analysis results for 4-hydroxialkenals outcomes. The table presents the results of the sensitivity analysis for 4-hydroxialkenals outcomes, showing both subgroup effects (see "Subgroup Effect" column) and overall effects (see "Overall Effect" column). The effect is presented before (see "No Exclusion" row) and after the exclusion of individual studies (see "Excluded Study" column and corresponding rows for the excluded studies). "Subgroup" column identifies the variable used for the subgroup analysis, and each entry corresponds to the subgroup associated with each result. "NA" indicates values that are not available after excluding individual studies, as subgroups were only considered when at least two studies were included in a subgroup. SMD: standardized mean difference. 95% CI: 95% confidence interval.

|  |  | Subgroup effect | | Overall effect | |
| --- | --- | --- | --- | --- | --- |
| Excluded study | Subgroup | SMD [95% CI] | p-value | SMD [95% CI] | p-value |
| No exclusion | Normal diet | -1.74 [-2.69, -0.79] | 0.0003 | -2.02 [-2.80, -1.25] | <0.0001 |
|  | High-fat diet | -2.60 [-3.94, -1.25] | 0.0002 |  |  |
| Shertzer *et al*., 2010 | Normal diet | -1.56 [-2.69, -0.42] | 0.0071 | -1.99 [-2.86, -1.12] | <0.0001 |
| Shertzer, 2010 | Normal diet | -1.54 [-2.67, -0.41] | 0.0075 | -1.98 [-2.85, -1.11] | <0.0001 |
| Shertzer *et al*., 2009 | Normal diet | -2.18 [-3.41, -0.94] | 0.0006 | -2.37 [-3.28, -1.46] | <0.0001 |
| Shertzer *et al*., 2010 | High-fat diet | NA | NA | -1.83 [-2.66, -0.99] | <0.0001 |
| Shertzer *et al*., 2009 | High-fat diet | NA | NA | -2.00 [-2.86, -1.13] | <0.0001 |

**Suppl. Table 24** Sensitivity analysis results for malondialdehyde outcomes. The overall effect (see "Overall Effect" column) is presented before (see "No Exclusion" row) and after the exclusion of individual studies (see "Excluded Study" column and corresponding rows for the excluded studies). "Condition" column identifies the dietary patter associated with each study. SMD: standardized mean difference. 95% CI: 95% confidence interval.

|  |  | Overall effect | |
| --- | --- | --- | --- |
| Excluded study | Subgroup | SMD [95% CI] | p-value |
| No exclusion | - | -0.49 [-1.32,0.34] | 0.2446 |
| Shertzer *et al*., 2009 | High-fat diet | -0.73 [-1.88, 0.42] | 0.2122 |
| Shertzer *et al*., 2009 | Normal diet | -0.68 [-1.94, 0.58] | 0.2917 |
| Shertzer, 2010 | Normal diet | -0.14 [-1.12, 0.85] | 0.7872 |
